# Solid-State Dewetting of Tungsten-Doped Vanadium Dioxide Nanoparticles: Implications for Thermochromic Coatings

**DOI:** 10.1021/acsanm.5c01247

**Published:** 2025-05-01

**Authors:** Samuel T. White, James R. Taylor, Ivan Chukhryaev, Silas M. Bailey, Joshua M. Queen, James R. McBride, Richard F. Haglund

**Affiliations:** † Department of Physics and Astronomy, Vanderbilt University, Nashville, Tennessee 37235, United States; # Vanderbilt Institute of Nanoscale Science and Engineering, Vanderbilt University, Nashville, Tennessee 37235, United States

**Keywords:** nanoparticle, dewetting, aggregation, phase-change, doping

## Abstract

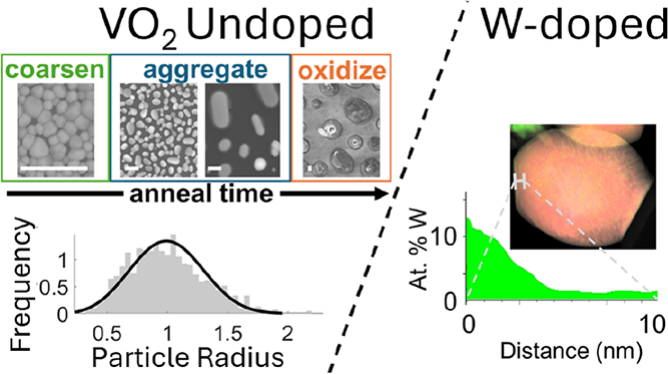

Doped vanadium dioxide (VO_2_) nanoparticles (NPs) have significant potential for applications requiring temperature-dependent emissivity, reflectivity, or transmission. Thermochromic coatings in particular enable energy-saving smart windows and passive thermal radiators but are subject to tight performance constraints. A major challenge is preparing uniform layers of NPs, over large areas, with controllable size distributions and transition temperatures (*T*_c_). We describe the growth and transition characteristics of randomly distributed undoped and W-doped VO_2_ NPs formed by solid-state dewetting. Sizes and size distributions are controlled by anneal time, as particles grow via Smoluchowski aggregation before oxidizing into V_2_O_5_; shapes are determined by the interfacial energies between VO_2_ (V_2_O_5_) and the silicon substrate. Tungsten dopants concentrate at the NP surface, increasing the energy barrier for and slowing the rate of dewetting, aggregation, and oxidization. Surprisingly, the doped NPs exhibit lower *T*_c_ and sharper hysteresis than comparably doped thin films. These results advance our capacity to engineer doped VO_2_ NPs, yield valuable insights into VO_2_–substrate interactions, and highlight the distribution of W-dopants in VO_2_ NPs.

## 1 Introduction

Vanadium dioxide (VO_2_) has long been a focus of interest in condensed-matter and materials physics because it undergoes a metal-to-insulator phase transition (MIT)^1^ near 70 °C. The sharp change in optical constants associated with this transition has enabled a range of devices,^2^ from ultrafast optical modulators^3^ to passive thermal control films. The latter is among the most mature VO_2_-based applications, providing the basis for energy-saving “smart windows” for terrestrial buildings^4,5^ and unpowered, low-weight thermal control coatings for spacecraft.^6^ These devices are often subject to strict constraints on transition temperature (*T*_c_) and optical properties^7^; smart windows, for example, require *T*_c_ near room-temperature for comfortable living spaces and high broadband transmission of visible light. Achieving the desired specifications while retaining strong infrared contrast between the hot and cold states continues to be a significant challenge, and new methods of manipulating these properties are required.

It is necessary to adjust *T*_c_ from its value of ∼68 °C in pure, unstrained, stoichiometric VO_2_. Substitutional dopants (such as tungsten and aluminum) which replace vanadium in the VO_2_ lattice, can affect *T*_c_ both by introducing local lattice strain and by contributing electrons or holes.^8^ Tungsten has been found to be particularly effective, suppressing *T*_c_ at a rate of up to −25 °C/at%W.^9^ Tungsten ions have a higher valence charge state (W^6+^ as compared to V^4+^), contributing two extra electrons to the lattice per W atom.^1^ The net effect is to stabilize the conductive rutile phase, driving down *T*_c_ (at thermal equilibrium) and increasing the speed of the transition (under ultrafast excitation).^10^ However, this generally comes at the cost of decreased hot–cold contrast.^11,12^ Annealing treatments can improve the transition behavior of W-doped thin films^13^ and nanoparticles^14^ by improving crystallinity, but still reduce optical transmission below acceptable limits. However, VO_2_ nanoparticles may overcome this doping-related loss of contrast, as one study has produced tungsten-doped VO_2_ nanoparticles embedded in an SiO_2_ matrix with *T*_c_ ≈ −20 °C without significant loss of contrast.^15^ Moreover, other dopants may also aid in improving optical transmission where that is required.^16^ Thus, the use of doped nanoparticles in smart windows appears to offer a technologically accessible route to maintaining both the requisite hot–cold transition *and* a comfortable optical transmissivity in living spaces.^17^

Producing high-quality, reproducible, doped VO_2_ nanoparticles with the desired transition properties on a large scale is a difficult task. Ongoing efforts continue to improve several well-established methods,^18−20^ each of which is subject to inherent limitations. Electron-beam lithography offers the most precise control of particle size, shape, and spacing,^21^ but is slow, expensive and does not scale to the sizes required for smart windows. Hydrothermal processes, on the other hand, are amenable to industry-scale production,^22^ and can yield a variety of different nanoparticle morphologies, but can be hard to reproduce, involve high-pressure processes, and the resulting particles can be difficult to adhere to a substrate.^19,20^ Moreover, questions remain about the fundamental physics relating transition behavior (*T*_c_, contrast, hysteresis width, transmission spectrum) to nanoparticle properties (size, shape, defects, dopants, and interfaces), especially as to how those properties might interact. Such studies are hampered by the cost and time of lithographic processing, and the difficulty of directly comparing hydrothermal nanoparticles to thin films.

**Scheme 1:**
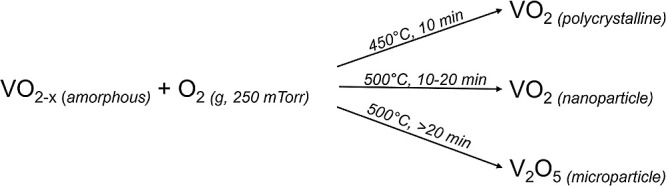
Amorphous, Non-Stoichiometric VO_2_ Films Are Annealed to Adjust Stoichiometry and Crystallinity (Upper), Produce Nanoparticles via Spontaneous Dewetting (Center), or Over-Oxidize into V_2_O_5_ (Lower) Certain conditions can lead to spontaneous dewetting, producing nanoparticulate VO_2_ (center).

Solid-state dewetting is a rapid and reliable method of producing single layers of VO_2_ nanoparticles, with scalability mainly limited by the film deposition method. Most VO_2_ thin film growth recipes involve a postdeposition anneal in an oxidizing environment to adjust stoichiometry and crystallinity and produce switching VO_2_, though overoxidation will lead to the formation of the more stable V_2_O_5_. Under certain conditions, this annealing process can cause VO_2_ to dewet from the substrate, producing nanoparticles (see Scheme 1). Although some progress has been made in controlling nanoparticle growth by this method (notably relating the particle size to film thickness),^23^ a more comprehensive understanding of the growth process and greater ability to control the nanoparticle characteristics are required to make it fully useful. Moreover, the role of W-doping on the nanoparticle formation process and resulting effects on transition behavior need clarification. Here, we investigate the effect of annealing time on the size and size distribution of VO_2_ nanoparticles. We show that after dewetting the nanoparticles coarsen, producing a particle size distribution (PSD) characteristic of Smoluchowski aggregation, which, apart from a scaling factor, depends only on particle diffusion behavior. Thus, anneal time provides an additional way to tune average nanoparticle size. We further show how this process differs in the case of W-doped nanoparticles. We find that tungsten doping fundamentally changes the dewetting/aggregation process, and that tungsten-doped nanoparticles have better hysteretic properties than corresponding thin films. These developments render solid-state dewetting a viable route to produce nanostructured VO_2_ films, providing a new tool to aid in the design and manufacture of thermochromic films with tailored thermal and optical behavior.

## 2 Experimental Section

Thin films of undoped VO_2_ were produced by atomic layer deposition (ALD) in a PicoSun R-200 system from water and tetrakis[ethylmethylamino] vanadium (TEMAV) precursors for 2500 pulse cycles. Each precursor was flowed at 150 sccm in 5 s pulses spaced 1 s apart. Substrate was held at 150 °C, TEMAV bottle at 70 °C, and valve block at 100 °C. ALD film thickness was measured by spectroscopic ellipsometry (JA Woollam M-2000VI) to be 111 ± 2 nm. All ALD samples were cut from two 4-in. wafers prepared in the same way. Tungsten-doped VO_2_ films (and corresponding undoped control films) were prepared with RF magnetron sputtering in an Angstrom Amod multimode deposition tool, at ∼280 W, in 6 mTorr Ar (20 sccm) and O_2_ (1 sccm). Films were deposited from metal W:V alloy targets, with 0:100 wt %, 5:95 wt % and 8:92 wt % compositions, for 450, 1000, and 630 s, respectively. Sputtered film thickness was measured with a Bruker Dektak 150 stylus profilometer to be 87 ± 4, 75 ± 4, and 110 ± 10 nm, respectively. For both ALD and sputtered films, the same postdeposition annealing process (450 °C, 250 mTorr O_2_, 10 min) was used to in order to produce switching VO_2_ thin films (Scheme 1). Dewetting was controlled by adjusting the temperature and time of the annealing process.

Films and nanoparticles at each set of anneal conditions were observed with a scanning electron microscope (SEM), using a Zeiss Merlin SEM with Gemini II Column, an in-lens detector, and ∼2 kV electron voltage. A few additional SEM images (Figure S8b and the blue- and orange-bordered images in Figure 5) were observed with an FEI Helios NanoLab G3 CX focused ion-beam (FIB) SEM, using a through-lens detector and 2 kV electron voltage. For undoped samples, grain/nanoparticle sizes and distributions were extracted using the open-source software ImageJ. From these data, normalized distributions of nanoparticle sizes were plotted, and other statistical analyses were performed using Matlab software. Contact angles were extracted from tilted SEM images, collected at an angle of 20.5° (for the 10 min sample) and 10° (for all others) above the plane of the surface, with the rotation axis parallel to the horizontal axis of the SEM images. Further details regarding image processing, particle counting, angle measurement, and tilt correction can be found in the Supporting Information, Section S1.

An X-ray diffractometer (XRD) (Rigaku Smart Lab, Cu Kα source) was used to perform standard θ–2θ measurements (scan 2θ from 20–80°, 0.01° steps, 10°/min). Optical hysteresis measurements were performed with a custom setup using a white-light tungsten halogen lamp (∼3000 K color temperature) and an amplified InGaAs photodiode. Transmission electron microscopy (TEM) and scanning transmission electron microscope energy dispersive spectroscopy (STEM-EDS) measurements were acquired on a Tecnai Osiris TEM/STEM operating at 200 kV. Drift-corrected STEM-EDS maps were acquired with a 1 nA probe current and rendered using Bruker Esprit version 1.9. Nanoparticle samples were prepared for TEM by scraping them off of the substrate with a razor blade, mixing them with IPA into a slurry, dipping a TEM grid into the slurry, and allowing it to dry. Some nanoparticles thus adhered to the grid.

## 3 Results and Discussion

**Figure 1:**
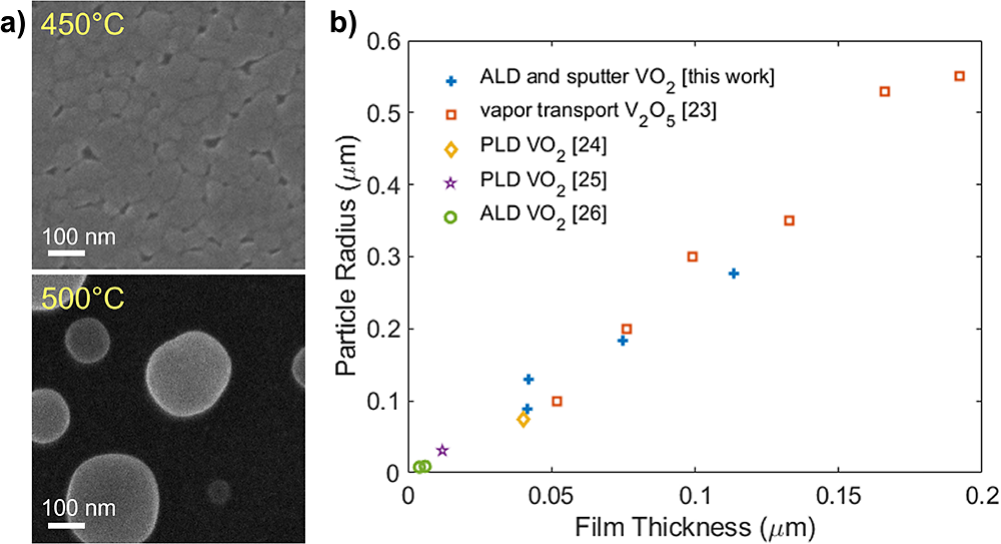
Polycrystalline thin films (a, upper) spontaneously dewet to form hemispherical nanoparticles (a, lower) when annealed at a higher temperature. (b) Across multiple studies producing films by different methods, the dewetting process results in nanoparticles with a radius that scales linearly with initial film thickness.

It has been shown that, under appropriate annealing conditions, solid VO_2_ thin films can dewet from Si (with native oxide) substrates to form roughly hemispherical nanoparticles,^23−26^ as exemplified in Figure 1a. Figure 1b compares nanoparticle sizes from these studies, and shows that the nanoparticle radius increases linearly with the thickness of the original film, as expected according to the theory of solid-state dewetting.^27,28^ This behavior is remarkably consistent across different film-deposition methods [atomic layer deposition (ALD), pulsed-laser deposition (PLD), sputtering, and vapor–solid transport], because it is governed entirely by the surface/interface energies of/between the substrate and film. Thus, dewetted nanoparticles can benefit from robust thin film growth techniques and their advantages (such as the conformal coating capabilities associated with ALD), and crucially can produce nanoparticles directly comparable to high-quality thin films. Also, established recipes for growing doped thin films will likely carry over to the production of doped, dewetted nanoparticles, as has been demonstrated with W-doped nanoparticles dewetted from W-doped films.^29^

**Figure 2:**
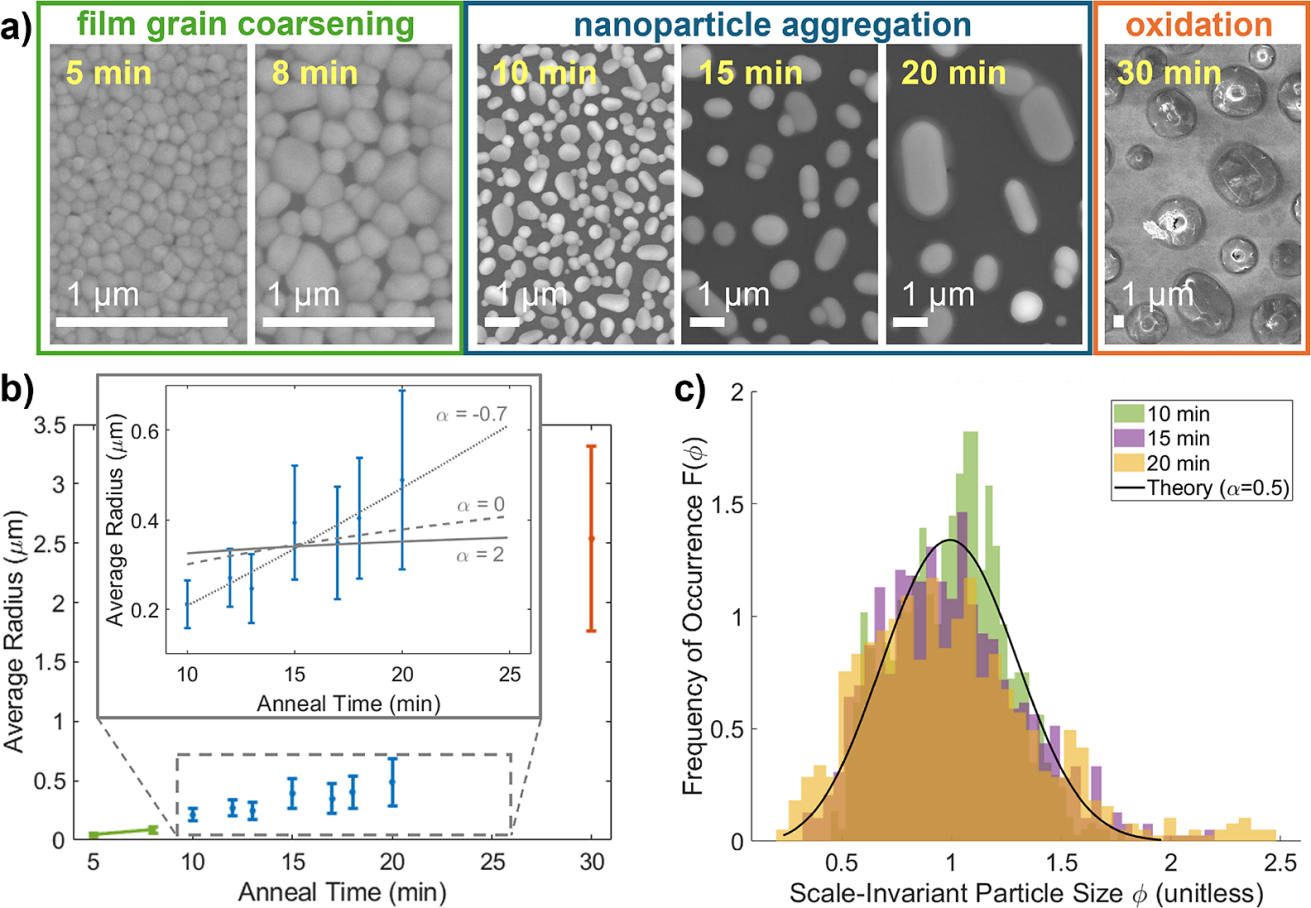
As anneal time increases (a), films first coarsen (green), then dewet into separate particles which grow larger over time (blue), and finally oxidize into V_2_O_5_(orange). Scale bar is 1 μm. The increase of particle size over time (b) and the resulting size distribution (c) can be qualitatively explained by Smoluchowski aggregation.

To investigate how the dewetting evolves as a function of annealing time, we first present SEM images of VO_2_ films prepared by atomic layer deposition (ALD) and annealed for progressively longer periods (Figure 2a). Starting as a 113 nm-thick polycrystalline film, the individual grains coarsen (from 5 to 8 min) until they separate into discrete particles (∼10 min). At longer times, these particles continue to grow (10–20 min). Eventually (30 min and beyond), the low-pressure oxygen environment oxidizes the nanoparticles fully into V_2_O_5_, drastically changing their shape and size as discussed further below. Figure 2b shows how average nanoparticle size varies with anneal time, with the three different regimes (grain coarsening, nanoparticle growth, and oxidation) represented by different colors (green, blue, orange).

The intermediate stage of particle growth is the regime in which we must work to produce nanoparticles, and determines both nanoparticle size and size distribution. There are two primary mechanisms by which particle growth may occur: transfer of individual atoms or small clusters from smaller particles to more stable larger particles (Ostwald ripening) or diffusion and coalescence of entire particles (Smoluchowski aggregation).^30−32^ The physics governing these processes is rich and complex; it is likely that both Ostwald ripening and Smoluchowski aggregation play a role in our observed particle growth, and which dominates may change throughout our growth process as the particles become larger and farther apart. However, the two mechanisms tend to produce qualitatively distinct particle size distributions (PSDs).^30−33^ Ostwald ripening results in a sharply peaked distribution with a broad tail to smaller particles, while Smoluchowski aggregation results in a broader distribution with a tail toward larger particles. Because our observed PSDs (Figure 2c) have the latter character, we rule out Ostwald ripening as the dominant mechanism. While other, noncoalescence driven coarsening models can also produce a Smoluchowski-like PSD,^34^ our SEM images often show pairs of particles in contact and apparently coalescing, thus we conclude that the Smoluchowski mechanism dominates under our conditions and present an analysis accordingly. Particle aggregation on these size and length scales would require a relatively large particle diffusion coefficient, on the order of 10^–8^ cm^2^/s. We are not aware of any published diffusion coefficients for this or a comparable system which we could use as a benchmark. However, large 3D atomic clusters have been observed to show unusually large diffusion coefficients on substrates with which they do not have an epitaxial relationship,^35^ as is the case here.

For aggregation-dominated coarsening, it is possible to derive a scale-invariant particle size distribution (PSD) *F*(φ) for long times, given only a few simplifying assumptions.^36,37^ First, that there are no collisions between three or more particles simultaneously (equivalent to neglecting higher-order terms of the Smoluchowski equation); although for short anneals (10 min) we can observe instances of 3 or more particles merging (Figure 2a), this is an increasingly good approximation at longer times. Second, that we use a time-independent, homogeneous kernel to the Smoluchowski equation; which is the case for cluster diffusion on a surface as long as there is no spatial correlation between particles. Sholl and Skodje^37^ show that, though spatial correlations begin to develop as the aggregation proceeds, this approximation nevertheless closely reproduces the correct PSD and rate of particle growth. Given these approximations, for three-dimensional particles constrained to a two-dimensional surface,
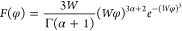
1where  is the scale-invariant particle size, Γ is the gamma function, and
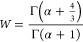
Note that *F*(φ) involves only one free parameter, α, the exponent which describes the mass-dependence of the diffusion coefficient, as
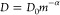
where *D* is the diffusion coefficient, *D*_0_ is a constant, and *m* is the particle mass. The exponent α is system-dependent, but typically varies from 0 to 2,^37^ and should approach 1 for large particles.^38^ The function *F*(φ) reproduces well the shape of our measured PSDs, as shown in Figure 2c.

The theory of Smoluchowski aggregation also predicts the time-dependence of the average particle radius *r̅*
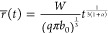
2where *q* is a geometrical factor accounting for the particle shape ( for a perfect hemisphere) and *b*_0_ is a scaling factor relating to initial conditions. Ideally, we could quantitatively measure α for our system by fitting our PSD to eq 1 or our radius-vs-time data to eq 2. However, we find that fitting the PSDs yields values of α varying from <0 to >1 (Supporting Information, Section S2), while fitting the radius-vs-time data (Figure 2b inset) yields α = −0.7 which would imply that larger particles diffuse faster than smaller particles. These discrepancies likely arise due to characteristics of the system not fully accounted for in the model assumption, most notably the development of spatial correlations over time,^37^ the possibility of multiple particles colliding simultaneously, the change in particle shape due to formation of V_2_O_5_, and the contribution of other coarsening mechanisms. Further discussion of this point can be found in the Supporting Information, Section S2.

**Figure 3:**
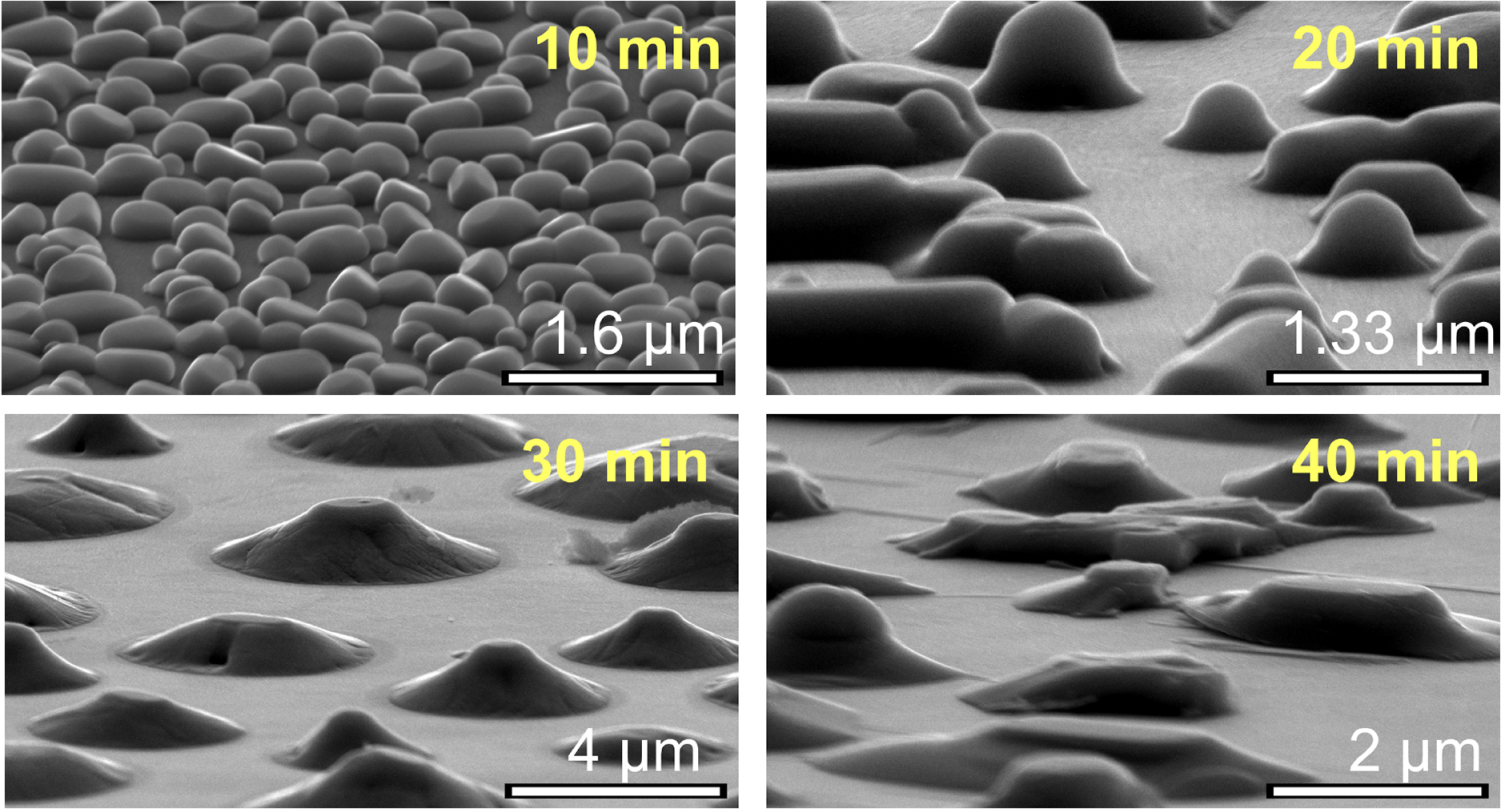
As anneal time increases beyond 10 min, another species begins to form. This species (likely V_2_O_5_) has a lower contact angle and forms a “skirt” around the nanoparticle. At long anneal times, V_2_O_5_ dominates over the VO_2_.

At longer anneal times (∼20 min and above), the particle morphology begins to change, exhibiting a “skirt” or “pedestal” around the particle perimeter, with a smaller contact angle θ on the substrate surface (Figure 3). This has previously been attributed to the formation of V_2_O_5_ at the nanoparticle surface due to overoxygenation.^24^ X-ray diffraction (XRD) measurements (Supporting Information, Section S3) and optical hysteresis measurements (Supporting Information, Section S7) show that characteristic VO_2_ lattice planes and switching behavior disappear at long times, indicating that a non-VO_2_ species is indeed formed. Raman spectroscopy (Supporting Information, Section S8) shows that, at sufficiently high anneal temperatures and/or long anneal times, the nanoparticles are fully oxidized into V_2_O_5_ droplets, supporting the idea that the “skirt” is formed of V_2_O_5_, though other vanadium oxides could be present at intermediate stages. At the highest temperatures probed (600 °C), another distinct species can be observed (Supporting Information, Section S5) with a crystalline, sheet-like structure. This is likely yet another vanadium oxide growing out of the V_2_O_5_ droplets, as observed in vapor-transport VO_2_ growth.^39^

Tilted SEM images (Figure 3) allow us to measure the contact angle between the nanoparticles and substrate. Contact angle θ obeys the Young–Laplace equation
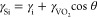
3where γ_Si_ and γ_VO_2__ are the surface energies of the Si substrate and VO_2_, respectively, and γ_i_ is the interface energy between them. Using literature values for γ_Si_ = 39 mJ/m^2^ (experimental, from ref (40), for p-type, B-doped Si(100) with native oxide, interpolated for Si with resistivity 0.1–1 Ω cm), γ_V_2_O_5__ = 90 mJ/m^2^ (experimental, from ref (41), average of tabulated values), and γ_VO_2__ = 320 mJ/m^2^ (theoretical, average from refs (42,43) for (110) rutile planes, which our XRD measurements in the Supporting Information show to be parallel to the substrate), we can calculate the interface energy at different time steps (Table 1). Since surface energies vary with temperature, and since no experimental surface energy values are available for VO_2_, these values are merely approximations, but they represent (to the best of our knowledge) the first approximations to the VO_2_/Si and V_2_O_5_/Si interface energies.

**Table 1:** Measured Contact Angles and Calculated Interface Energies for VO_2_ and V_2_O_5_ Nanoparticles on Si

	10 min	15 min	20 min	30 min	40 min
θ (°)	122 ± 2	47 ± 2	42 ± 2	33 ± 1	29 ± 2
γ_i_ (mJ/m^2^)	210^a^	–22^b^	–28^b^	–36^b^	–40^c^

a For VO_2_.

b For intermediate mixed phase.

c For V_2_O_5_.

Uncertainties on contact-angle measurements represent one standard error of the mean (refer to Supporting Information, Section S1, for details on the contact angle measurement). The contact angle is initially large (122°), corresponding to its value for VO_2_ on Si. Using γ_VO_2__ = 320 mJ/m^2^, we calculate the VO_2_/Si interface energy to be γ_i_ ≈ 210 mJ/m^2^. As annealing proceeds and V_2_O_5_ begins to form, the contact angle decreases, asymptotically approaching its value for V_2_O_5_ on Si (29°). Using the V_2_O_5_ surface energy (γ_V_2_O_5__ = 90) we calculate the V_2_O_5_/Si interface energy to be γ_i_ ≈ −40 mJ/m^2^. Intermediate values for γ_*i*_ are calculated using the V_2_O_5_ surface energy since the contact angles and SEM images suggest the edge species is V_2_O_5_; however, due to the presence of both VO_2_ and V_2_O_5_ in these particles, the calculated contact angles do not truly represent either interface.

**Figure 4:**
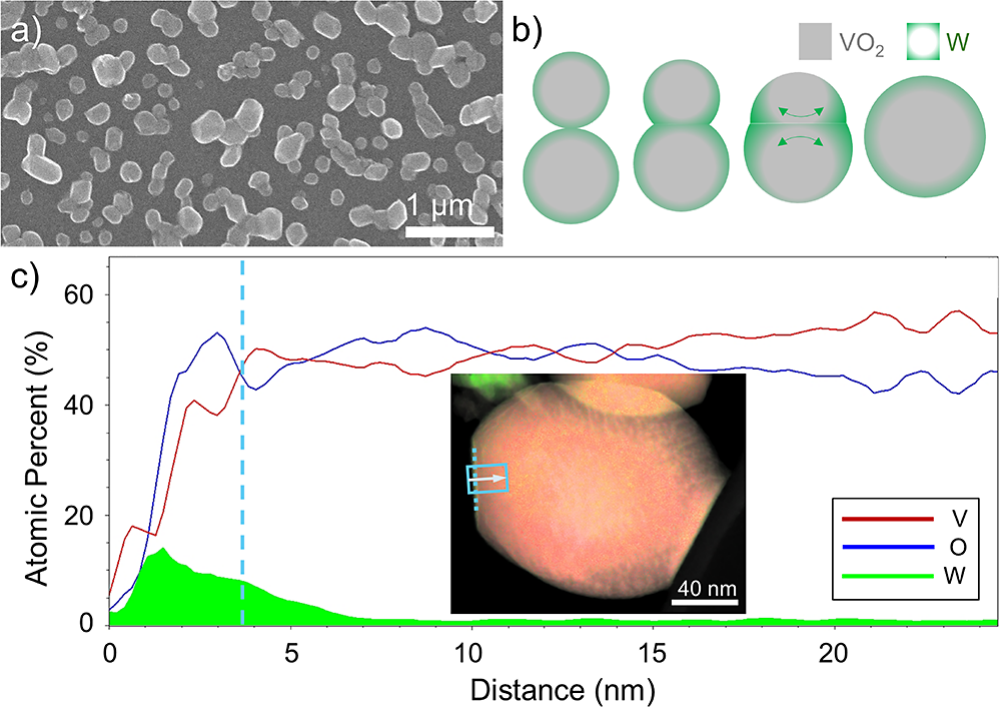
Tungsten-doped, dewetted nanoparticles (a) are more strongly faceted and tend to group into clusters. The energy barrier for two doped particles to coalesce is increased because the W-dopants must be extruded from the interfacial region (b) in order to preserve the segregation of dopants at the nanoparticle surface, as observed by energy-dispersive spectroscopy in TEM (c). Percent abundance of V (red), O (blue), and W (green) is shown along the line cut indicated by the gray arrow (inset), averaged over the width indicated by the blue rectangle. Blue dashed lines indicated the location of the particle edge.

Tungsten-doped VO_2_ films also form nanoparticles through dewetting, but with two significant qualitative differences (Figure 4). First, W-doped films require a higher temperature and longer anneal times to dewet; undoped films dewet in 10 min at 500 °C, but W-doped films require at least 600 °C and 20 min before significant dewetting occurs (see Supporting Information, Section S4 for comparison of doped and undoped films annealed at different temperatures, and Section S5 for analysis of other species formed at high anneal temperatures). Second, unlike the well-separated, nearly hemispherical nanoparticles that form from undoped films, W-doped particles are distinctly faceted and clustered together (Figure 4a). Taken together, the higher temperature, longer time, and clustering behavior suggest that the presence of tungsten increases the energy required and slows the rates of dewetting, particle diffusion, and particle coalescence.

Energy dispersive X-ray spectroscopy (EDS) in a scanning transmission electron microscope (STEM) allows us map out the distribution of W atoms in some of these nanoparticles (Figure 4c). We find that the W concentration is highest at the outside edge (the first ∼5–10 nm) of the nanoparticles, and decreases toward the center (additional examples in other nanoparticles, and a reference measurement on an undoped nanoparticle, are presented in the Supporting Information, Section S6). This is an example of dopant segregation arising from minimization of both strain and electrostatic energy; similar behavior has been observed in other doped metal-oxide nanoparticles such as SnO_2_.^44^ Based on this observation, we conclude that the slower particle growth and the clustering behavior in W-doped films result in part from the W-rich boundary layer on the nanoparticle surface (Figure 4b), which retards grain boundary motion and particle coalescence by both thermodynamic and kinetic considerations.^45^

On the one hand, coalescence of two crystalline particles generally involves an energy barrier associated with the atomic rearrangement necessary to incorporate one into the crystal lattice of the other^46^; intuitively, the W-rich surface layer (representing a low-energy configuration) increases this barrier by at least the amount of energy necessary to move those W atoms from the interface to the surface of the new, larger particle. Thus, a higher temperature is required to overcome this increased energy barrier. Moreover, in certain systems the presence of solute atoms segregated to grain boundaries has been shown to lower the grain boundary energy,^47^ disfavoring grain (particle) coalescence necessary for dewetting (coarsening) of films (nanoparticles). At the same time, even at the requisite higher temperature such that energetic considerations are satisfied, dewetting and particle coalescence is slowed by the solute drag effect—the retardation of grain boundary motion in the presence of segregated solute atoms due to the slower diffusion rates of those solute atoms through the lattice.^48,49^ Thus, the segregation of tungsten to grain boundaries (particle surfaces) both thermodynamically disfavors and kinetically impedes dewetting (particle aggregation). As a result, at any given point during the aggregation process, there are likely to be more particles that have met, but not fully merged into a single particle (hence the “clustering” noted above). This spontaneous formation of a dopant-rich shell and consequent reduction of particle coarsening has been observed in other systems and employed to help stabilize small particle sizes in SnO_2_ nanoparticles.^44^ It seems likely that the presence of tungsten also slows the rate of particle diffusion across the substrate; a quantitative analysis of particle diffusion rates and how they are affected by dopant segregation is beyond the scope of this work, and would be an interesting area for further study.

**Figure 5:**
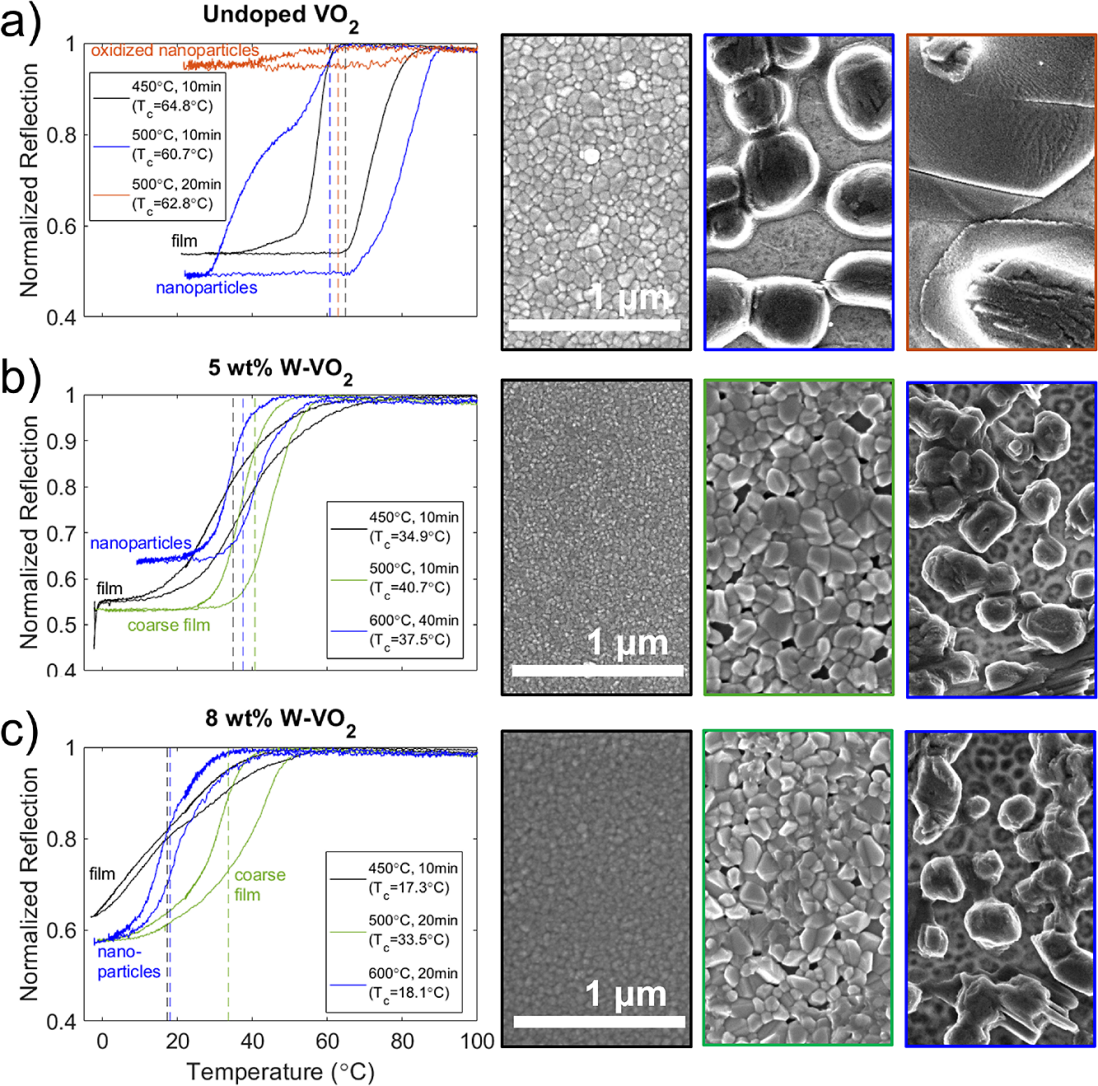
In undoped VO_2_ (a) the hysteresis loop for nanoparticles (blue) has greater width (due to fewer nucleating defects) and slightly better contrast (possibly due to changes in scattering) than for a corresponding thin film (black). Further annealing begins to change VO_2_ into V_2_O_5_, reducing contrast (orange). In W-doped VO_2_ (b, c), films (black) have poorer hysteresis shape. Annealing at higher temperatures (green) improves this, but at the cost of increased *T*_c_. Formation of doped nanoparticles (blue) restores the low *T*_c_ while retaining the better hysteresis shape.

Finally, Figure 5 compares the phase transition behavior in doped and undoped samples annealed at different conditions to produce small-grained films (black), coarse-grained films (green), nanoparticles (blue), or nanoparticles that have largely been oxidized into a non-VO_2_ species (orange). Non-normalized versions of these data, and additional optical hysteresis curves for an undoped VO_2_ film at various stages of dewetting can be found in Supporting Information, Figure S12. For undoped VO_2_ (Figure 5a), nanoparticles have a wider hysteresis loop than corresponding thin films. This is a known effect in VO_2_ nanoparticles due to their lower density of transition-nucleating defects.^50^ At longer anneal times, the width of the hysteresis loop is maintained, but the contrast decreases as more VO_2_ is oxidized into V_2_O_5_. For W-doped VO_2_ films (Figure 5b,c), the hysteresis loop has a shallow slope, and at high doping levels (Figure 5c) an unusual shape. Annealing at higher temperatures (green) produces steeper hysteresis, likely due to improved crystallinity of the grains and more uniform distribution of the dopant atoms. While the shape of the hysteresis loop is improved, a sign of higher-quality VO_2_, the transition temperature (broken vertical line) increases slightly. Upon the formation of nanoparticles, the hysteresis loop does not broaden appreciably (as it does in the undoped case), because the W-dopants themselves form transition-nucleating defects. However, the transition temperature decreases slightly, while retaining the better shape and contrast of the coarse-grain films (most clearly seen for the highest W concentration). Thus, nanoparticle formation helps to counter the degradation of switching behavior caused by heavy W doping, making it possible to achieve good switching at lower temperatures than is possible in thin films. This qualitatively agrees with observations of W-doped VO_2_ nanoparticles formed by ion implantation^15^; though that study achieved much lower transition temperatures at similar doping levels, implying that encapsulation in an SiO_2_ matrix also played an important role in their results. Switching contrast is another important metric for device applications. Contrast falls within a narrow range (roughly 40–50%) for all samples measured (except the case of heavy oxidation where it is severely reduced). In thin films, contrast decreases steadily as W concentration increases; for nanoparticles there is not a clear trend, and the contrast is sometimes better or worse than that of the corresponding thin film. Because switching contrast depends not only upon intrinsic VO_2_ properties but also upon film thickness (via thin-film interference), surface coverage, and nanoparticle size (via scattering), these small observed changes are likely due to sample-to-sample variation or intrasample inhomogeneity. Fully understanding the details of contrast variation will require a more thorough investigation of the scattering behavior of these nanoparticles in the metallic and insulating states.

## 4 Conclusions

Vanadium dioxide NPs are a promising active component of thermochromic films for passive temperature control. We have shown that solid-state dewetting of doped and undoped VO_2_ thin films is a viable method of rapidly producing large-area, single-particle-thick films of such nanoparticles, and we have investigated the physics of their formation and the changes introduced by doping. The average nanoparticle size can be adjusted not only by varying initial film thickness, but also by adjusting the anneal time. As anneal time is increased, the nanoparticles coarsen, resulting in a characteristic particle-size distribution. The observed PSD is well-described by a coalescence-driven Smoluchowski aggregation process. Fully elucidating the nuances of the coarsening mechanism would be a fruitful avenue for further studies, especially in situ measurements. We also report estimates of the interfacial energies between VO_2_/V_2_O_5_ and Si with a native oxide. These insights will be crucial to the design of actual devices based on dewetted-VO_2_ nanoparticles. Despite the potential of W-doped VO_2_ nanoparticles to enhance the performance of thermochromics, there has been little study on the dewetting process in W-doped VO_2_ films. We have shown that nanoparticles formed by this method have a particularly high W-concentration on their surfaces, leading to an increased energy barrier to particle aggregation. It is remarkable that the higher temperatures and longer times required for W-doped films to dewet cause undoped films to be completely oxidized into V_2_O_5_, implying that the W-doped films are more stable in an oxidizing environment; possibly the W-rich layer at the nanoparticle surface acts to protect the VO_2_ inside by reacting with atmospheric oxygen itself. Since VO_2_ films exposed to atmospheric conditions have a tendency to transform into V_2_O_5_ over time, W-doped films may have a longer shelf life than their undoped counterparts, an important consideration for their commercial applicability. Though a long-term study of film stability is beyond the scope of this work, preliminary tests (Supporting Information, Section S9) show slightly better stability in the W-doped samples. Finally, we have shown that nanoparticle formation can improve the hysteretic behavior of heavily doped VO_2_, an important advancement for applications requiring well-defined switching behavior at low temperatures. Understanding these results is an important step to improving the fabrication of dewetted, W-doped nanoparticles, and, more importantly, to understanding the effects of W-dopants on the VO_2_ phase transition itself. Thus, we anticipate this method and these insights to be a valuable tool in the further development and optimization of thermochromic films, improving prospects for unpowered temperature control in a variety of environments.

## Supporting Information Available

Experimental methods and materials; statistics and curve fits for the particle size distributions; XRD measurements as a function of anneal time; additional images of samples annealed at different times and temperatures; electron microscope analysis of additional species formed at long anneal times; additional TEM data, including EDS spectra and maps; additional hysteresis measurements for undoped VO_2_ at different anneal times and non-normalized versions of hysteresis data from Figure 5; Raman spectra showing the presence of VO_2_ or V_2_O_5_ different samples; and hysteresis measurements performed after 19 months of aging (PDF)
