# Supplementary material for: Solid-State Dewetting of Tungsten-Doped Vanadium Dioxide Nanoparticles: Implications for Thermochromic Coatings
Source: ACS Appl Nano Mater. 2025 May 1;8(19):9972–80. doi: 10.1021/acsanm.5c01247 (PMC12090180; doi:10.1021/acsanm.5c01247)
Supplement: Supplementary file 1 — an5c01247_si_001.pdf [file an5c01247_si_001.pdf]

SUPPORTING INFORMATION FOR Solid-State Dewetting of Tungsten-Doped Vanadium Dioxide Nanoparticles: Implications for Thermochromic Coatings

Samuel T. White,[*,†,‡] James R. Taylor,[†,¶] Ivan Chukhryaev,[†,§] Silas M. Bailey,[†,∥] Joshua M. Queen,[†,⊥] James R. McBride,[#] and Richard F. Haglund, Jr.[*,†]

†*Department of Physics and Astronomy, Vanderbilt University, Nashville, TN, 37235, United States*

‡*Current Affiliation: National Research Council Postdoctoral Associate at the Naval Research Laboratory, Washington, DC, 20375*

¶*Current Affiliation: Wyant College of Optical Sciences, University of Arizona, Tucson, AZ, 85721, United States*

§*Current Affiliation: Department of Applied Physics and Applied Mathematics, Columbia University, New York, NY, 10027, United States*

∥*Current Affiliation: Department of Physics, Columbia University, New York, NY, 10027, United States*

⊥*Current Affiliation: Department of Physics, NC State University, Raleigh, NC, 27695, United States*

#*Vanderbilt Institute of Nanoscale Science and Engineering, Vanderbilt University, Nashville, TN, 37235, United States*

E-mail: samuel.t.white26.ctr@us.navy.mil; richard.haglund@vanderbilt.edu

S1: SEM Image Analysis

To measure particle size distributions in SEM images, the raw images were first converted to greyscale, then converted to black and white using a threshold function, and finally inverted to yield black particles on a white background. ImageJ's built-in particle analysis tool was then used to detect, count, and measure the area of each particle (after scaling the image based on a scale bar produced by the SEM software). For anneal times under 15 minutes, many grains/particles were still in contact with neighboring particles, though divided by a clear grain boundary. In order for the software to distinguish such grain/particles, it was necessary to mark some of the boundaries manually with a black line. Figure S1 demonstrates this markup process, with the raw (or purely computer-processed) images on the left, and the manually adjusted images on the right. The effective radius of each particle is calculated from its measured area by assuming each grain/particle is a spherical cap (with a circular cross-section).

Contact angles were measured manually using an angle measurement tool in ImageJ, measuring the tangent of the nanoparticle relative to the horizontal at its corner (examples of these angle measurements are shown in Figure S2a). For each anneal time, angles are measured for multiple particles and averaged to give the reported contact angle. Since these images are not collected exactly edge-on, but at a slight tilt, a geometric correction must be applied to find the actual contact angle. If the true contact angle is θ, the measured angle is θ', and the tilt of the viewing axis above the substrate plane is ϕ, then

$$\theta = \arccos\left(\frac{\cos\theta' + \cos\phi - 1}{\cos\phi}\right) \tag{S1}$$

To derive this correction, consider a sphere of radius R centered at the origin of a coordinate system: $R^2 = x^2 + y^2 + z^2$. Let the z-axis represent the viewing angle of our SEM image. The nanoparticle is a spherical cap formed by the intersection of this sphere with the substrate plane. Let this plane be parallel to the x-axis, inclined relative to the z-axis

by the viewing angle ϕ, and a distance $d = R - h$ from the origin. Then the plane can be represented $y = \frac{R-h}{\cos\phi} - \frac{\sin\phi}{\cos\phi} z$ for all x, the spherical cap has height h above the substrate plane, and the vector $R\hat{n} = R\sin\phi\hat{z} + R\cos\phi\hat{y}$ is the radial vector normal to the substrate plane. Figure S2b shows a schematic of this scenario.

When viewed along the substrate plane (Figure S2c), the particle appears as the section (height h) of a circle (radius R). When viewed along the z-axis, however, it appears as the projection of the 3D speheric section onto the xy-plane. The intersection of the sphere and substrate plane is given by $R^2 = x^2 + (R-h)^2 + \frac{1}{\sin^2\phi}[y - (R-h)\cos\phi]^2$, a circle in 3D space, but an ellipse projected onto the xy-plane. This ellipse has a centerline $y = (R-h)\cos\phi$. The projection of our particle (Figure S2d) is then bounded below by the bottom half of this ellipse and above by a circular section with radius R and height $h' = R - (R-h)\cos\phi$.

The contact angle of a circular section is given by $\theta = \arccos\frac{h}{R}$. Applying this to the actual and apparent particle cross-sections, and using the above equation relating h and h', we can solve for the actual contact angle θ in terms of the apparent angle θ', yielding Equation S1 above.

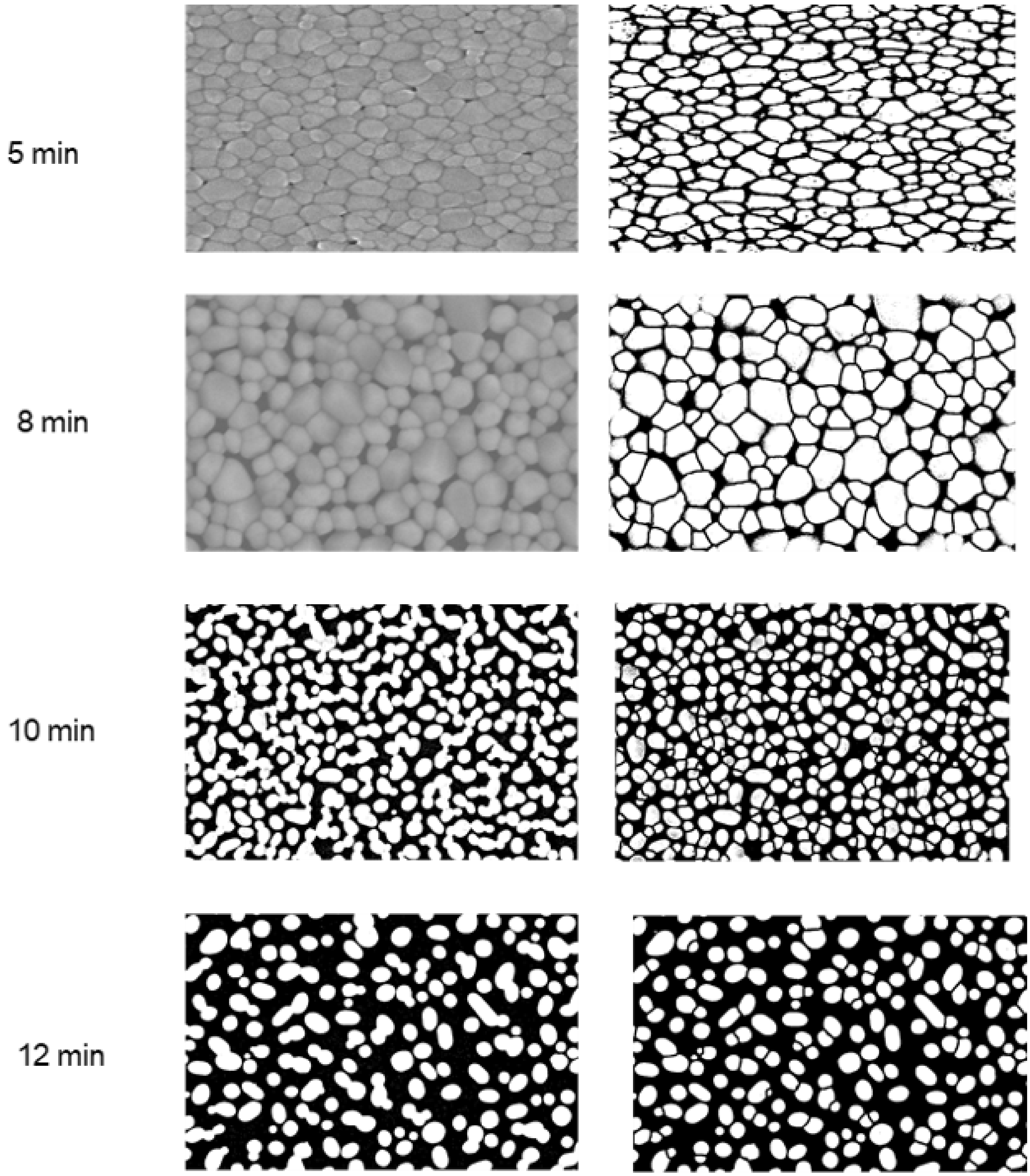

Figure S1: In samples with grains/particles that are in contact, the grain boundaries are often missed by the thresholding and particle analysis functions, making it necessary to adjust the images by hand. Raw SEM images (5 and 8 min) and thresholded images (10 and 12 min) are shown on the left; their adjusted counterparts on the right.

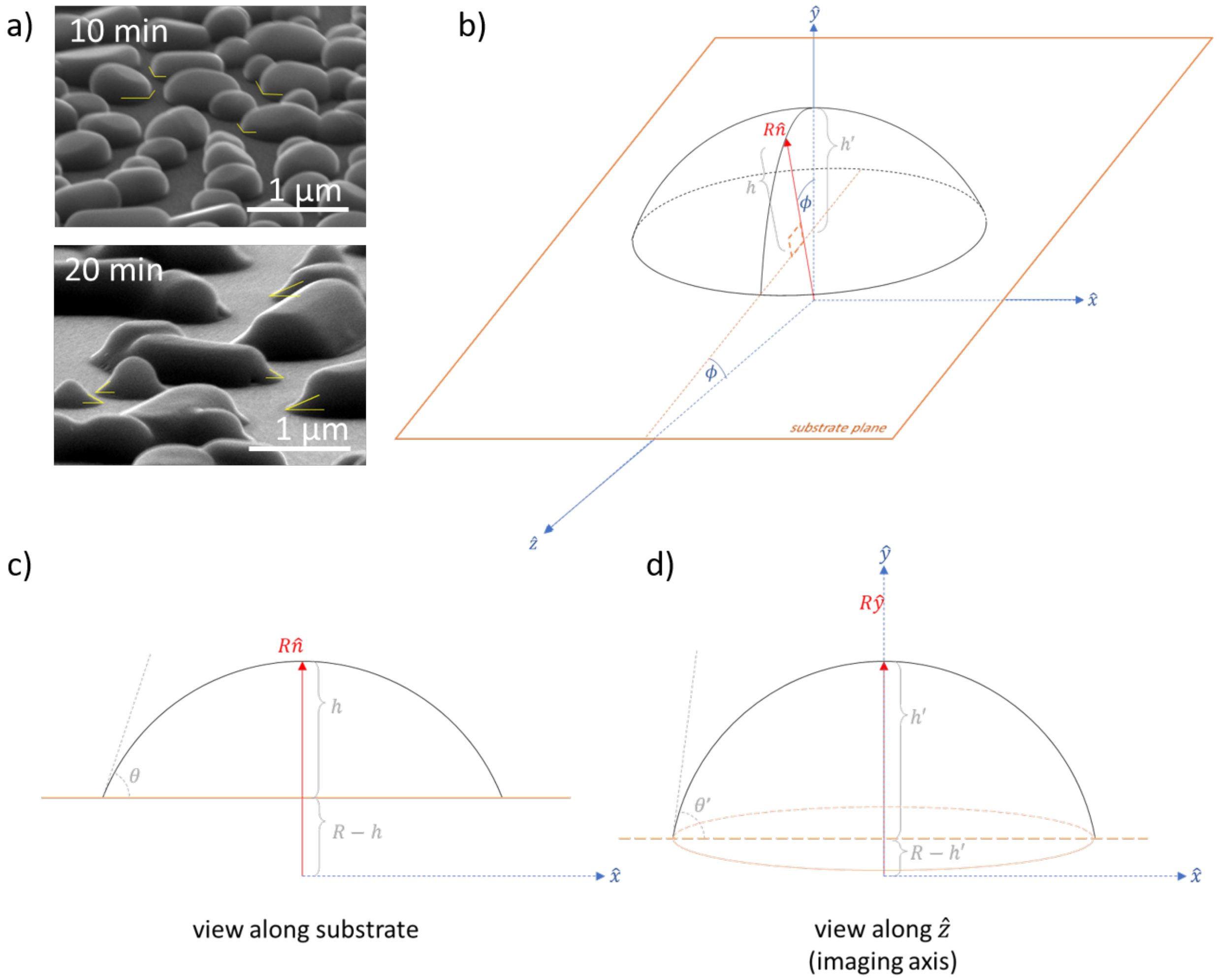

Figure S2: To extract contact angles, the angle between the horizontal and the particle tanget was measured at its point of contact with the surface, for several particles at each time step (a, yellow marks). The particles can be modeled as sections of a sphere cut by the plane of the substrate surface (b). When viewed along a direction not parallel to the surface (c), the apparent height of that section changes, affecting the measured contact angle.

S2: PSD Fitting Results

Particle size distributions (PSDs) were extracted from SEM images for VO_2 films annealed at different lengths of time (5-30 min), and used to calculate average particle radius $\bar{r}$ and standard deviation $\Delta\bar{r}$ (plotted in the main text, Figure 2b). The diffusion coefficient exponent α and 95% confidence interval $\Delta\alpha$ are extracted by fitting each PSD to the theoretical distribution $F(\phi)$ using the "maximum likilhood estimates" function in MATLAB, with α as a free parameter. The measured PSDs and best fit $F(\phi)$ curves are plotted in Figure S3a. For comparison, they are plotted with $F(\phi)$ for a constant $\alpha = 0.5$ in S3b. To quantify how well each experimental PSD matches the shape of the theory, we compare $\Delta\bar{r}$, skewness, and kurtosis to $F(\phi)$ with the corresponding value of α. The results are summarized in Table S1. Note that since $\Delta\alpha$ is a result of the fitting process and not a characteristic of the distribution $F(\phi)$, and $F(\phi)$ depends on the normalized size ϕ rather than the actual size $\bar{r}$, $\Delta\alpha$ and $\bar{r}$ are not reported for $F(\phi)$.

As noted in the main text, α varies considerably across the different data sets, though we don't expect it to change drastically for a given system (VO_2 on Si at 500°C). Figure S3c shows the extracted values of α as a function of anneal time. Qualitatively, a higher value of α corresponds to a sharper, narrower distribution, and vice versa (see Figure S3d). At short (< 10 min) and long (> 20 min) anneal times, $\alpha \approx 0.3$, but during the timeframe where particle aggregation dominates (10-20 min), α appears to decrease from ~ 1 to ~ -0.2. On the other hand, fitting the radius vs. time data (main text, Figure 2b) yielded a value of $\alpha \approx -0.7$. To confirm this fit value, the slope of a log-log plot of radius vs time (Figure S3e) also yields $\frac{1}{3(1+\alpha)} = 1.16$, or $\alpha \approx -0.7$. These discrepancies can be explained in terms of deviations of our system from the model. Initially, the PSD will be similar to that of the as-deposited film, and governed by the physics of film deposition rather than by the aggregation. At short times, when particles are small and closely packed, the probability of more than two particles colliding simultaneously is high; while at longer times, the formation of V_2O_5, which both wets Si better and has a lower density, causes the particle radii to be

Table S1: Fit results and statistical analysis of PSDs.

PSD	α	$\Delta\alpha$	$\bar{r}$	$\Delta\bar{r}$	Kurtosis	Skewness
5 min	0.23	0.10	0.04	0.01	2.9	0.27
F(ϕ)	0.23	-	-	0.01	2.6	0.11
8 min	0.35	0.16	0.09	0.03	3.3	0.61
F(ϕ)	0.35	-	-	0.03	2.7	0.09
10 min	0.99	0.15	0.21	0.05	2.5	0.10
F(ϕ)	0.99	-	-	0.05	2.8	0.04
12 min	1.12	0.22	0.27	0.06	2.7	0.05
F(ϕ)	1.12	-	-	0.06	2.8	0.04
13 min	0.29	0.11	0.25	0.08	11.0	1.99
F(ϕ)	0.29	-	-	0.08	2.6	0.10
15 min	0.25	0.10	0.39	0.13	3.2	0.57
F(ϕ)	0.25	-	-	0.12	2.6	0.11
17 min	0.02	0.13	0.35	0.12	3.2	0.38
F(ϕ)	0.02	-	-	0.12	2.5	0.13
18 min	0.18	0.10	0.40	0.13	3.1	0.58
F(ϕ)	0.18	-	-	0.13	2.6	0.11
20 min	-0.17	0.07	0.49	0.20	4.1	0.86
F(ϕ)	-0.17	-	-	0.18	2.4	0.15
30 min	0.37	0.17	2.54	0.78	2.7	0.32
F(ϕ)	0.37	-	-	0.76	2.7	0.09

larger than they would be otherwise. Both of these effects will cause the measured particle radii to grow faster than our model would predict, explaining the poor value of α obtained from fitting the radius vs time data. The extracted values of α at intermediate times (~13-18 min) are probably the best approximation, and suggest that $\alpha \approx 0.2 - 0.3$ for this system.

The standard deviations $\Delta\bar{r}$ agree very well between the actual distributions and the best-fit PSDs, showing that the theory well describes the spread of particle sizes we observe. Kurtosis measures how large the "tails" of the distribution are relative to a Gaussian (for which kurtosis = 3); a sharper peak with heavy tails has a larger kurtosis, and vice-versa. All of our calculated distributions $F(\phi)$ have a sub-Gaussian kurtosis (slightly lighter tails than a Gaussian); in each of the measured distributions, kurtosis is slightly higher. Skewness is a measure of asymmetry in a distribution, zero for a Gaussian distribution. $F(\phi)$ has a positive skewness reflecting the heavier tail toward larger radii. In the measured distribu-

tions, skewness is even higher than predicted, and varies sample-to-sample more than the kurtosis. The deviation of skewness and kurtosis from the expected may be due in part to the failures of our assumptions cited above; but since the skewness and kurtosis (unlike the fitted values of α) don't follow any clear trends with anneal time, their variation is probably due more to noise in the data. Performing this analysis on a larger sample size could improve the statistics and clarify these differences. The 13-min sample is clearly an outlier, with exceptionally large kurtosis (11) and skewness (1.99). Its unusual shape can be easily seen in the distribution (Figure S3a and S3b, green curve) with a sharp peak below $\phi = 1$. This is again attributed to random variation, resulting in an unusual concentration of smaller-than-average particles in the sampled area.

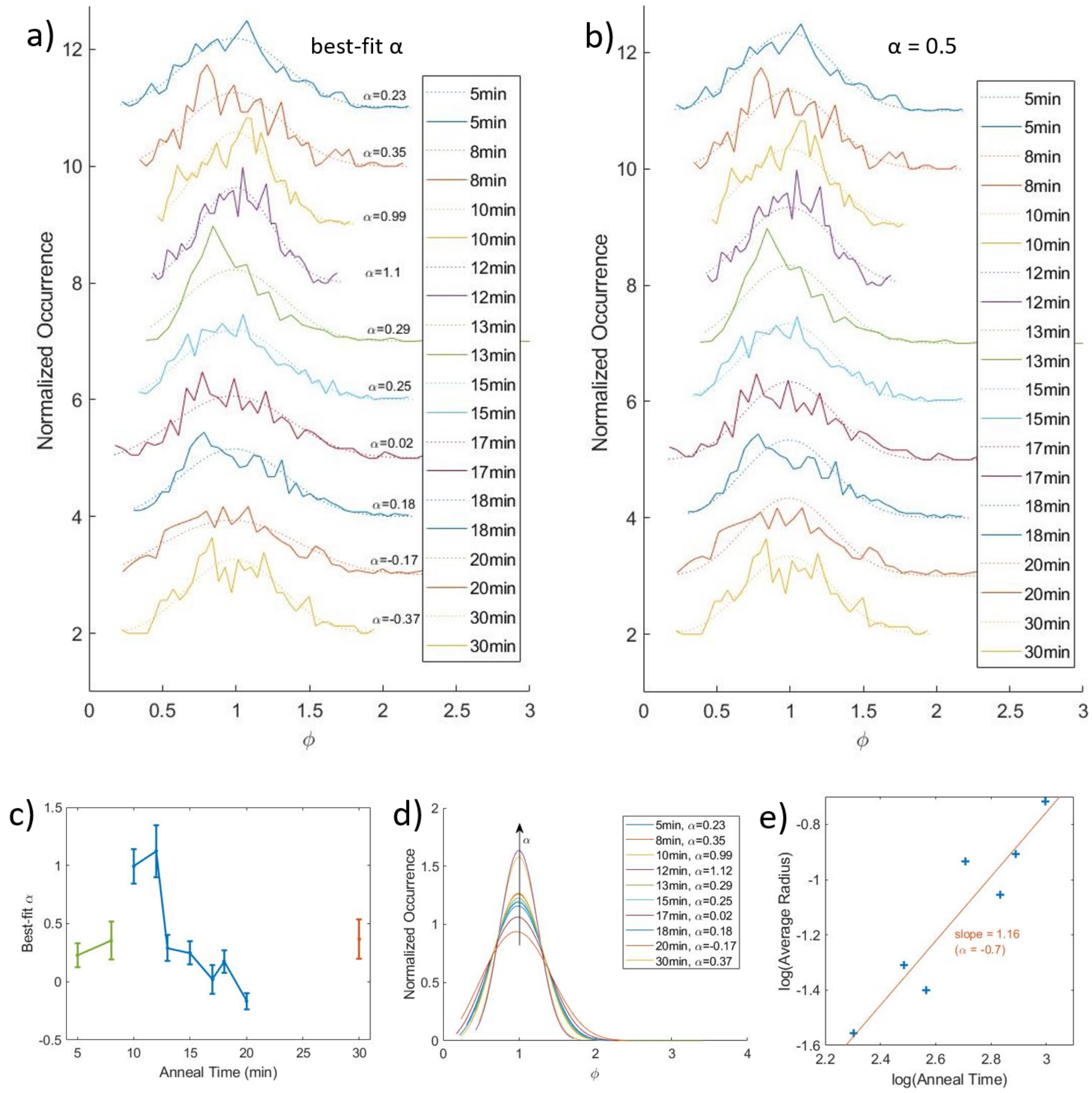

Figure S3: The measured particle size distributions can be well fit by the predicted funtion $F(\phi)$ with α as the only fit parameter (a). For comparison, using a constant value of $\alpha = 0.5$ qualitatively reproduces the PSD (b). The values of α obtained by fitting experimental PSDs vary over time due to physical effects not accounted for in the model (c). A higher value of α corresponds to a narrower distribution (d). A log-log plot of particle radius vs anneal time (e) produces the same value of $\alpha \approx -0.7$ as the curve fit presented in the main text.

S3: XRD measurements on VO_2 nanoparticles

X-ray diffraction spectra (Figure S4a) show peaks corresponding to VO_2 (marked in purple) as well as to the silicon substrate (black) and the steel sample stage (grey). Only one VO_2 plane can be identified: the XRD peaks at $2\theta = 25.2°$, $2\theta = 27.9°$, and $2\theta = 57.6°$corresponding to the $\{011\}$ family of planes in M1-phase VO_2. This suggests that the nanoparticles tend to orient with the $\{011\}$ planes parallel to the substrate; this is the lowest-surface-energy plane for VO_2, and commonly forms bounding facets on VO_2 crystals.[1] However, as this is the strongest reflection in VO_2, small proportions of other orientations could be present and below the noise level. The absence of corresponding peaks for M2- and R-phase VO_2 ($\{20\bar{1}\}_{M2}$ at $2\theta = 27.4°$, $\{201\}_{M2}$ at $2\theta = 28.3°$, and $\{110\}_R$ at $2\theta = 27.7°$) shows that we have phase-pure M1 VO_2.

This $\{011\}_R$ peak evolves as a function of anneal time (Figure S4b). Peak height, center position, and breadth (Figure S4c) are extracted by fitting to a sum of two Lorentzian peaks, (peak splitting arises from the presence of Cu $K_{\alpha 1}$ and $K_{\alpha 2}$ lines in the X-ray source). Solid lines (black error bars) represent the lower-angle peak, and dotted lines (grey error bars) the higher. Initially, the peaks are very weak and broad (overlapping to the point of being indistinguishable), due to poor crystallinity and small grain size of as-deposited VO_2. At intermediate anneal times, the intensity increases as the VO_2 becomes more crystalline and as the crystalline nanoparticles grow larger. At long times, the intensity decreases and peaks broaden (becoming indistinguishable) again, as the VO_2 is transformed into V_2O_5. The absence of any additional XRD peaks corresponding to V_2O_5 suggests that the V_2O_5 formed by oxidation is highly amorphous.

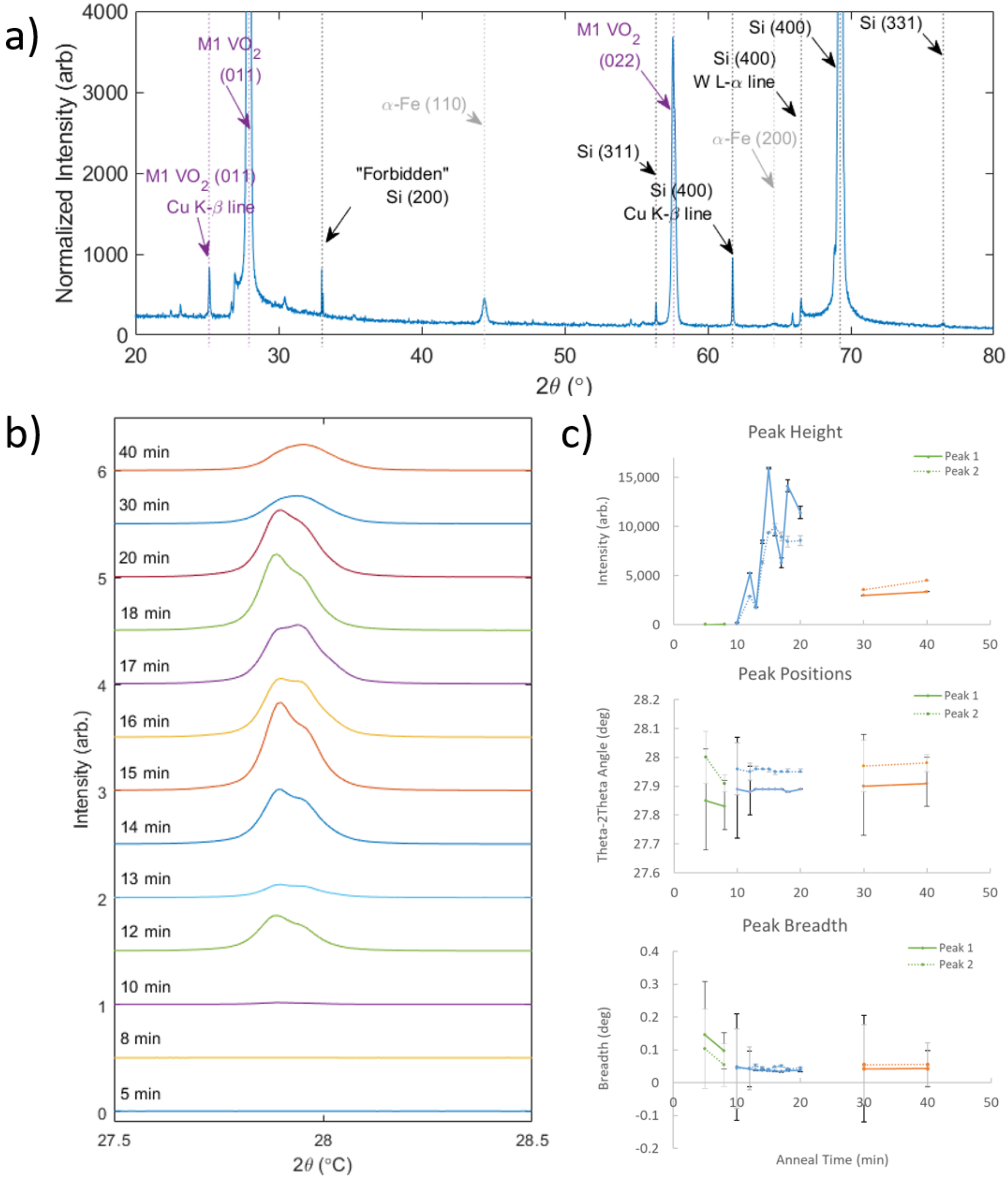

Figure S4: XRD spectra (a) show the presence of (011)-oriented M1-phase VO_2. The corresponding peak changes as a function of anneal time (b), revealing information about the change in crystallinity over time (c).

S4: Undoped and W-doped films annealed at different temperatures and times

Figures S5, S6, and S7 demonstrate the results of different annealing conditions tested on undoped, 5 wt% W-doped, and 8 wt% W-doped sputtered films. Annealing at 450°C for 10 minutes is the standard annealing protocol to produce switching thin films, and in each case results in a polycrystalline film with small, densely packed grains. In undoped samples, dewetting begins to occur at 500°C, 10 min; at 20 min, the nanoparticles have grown through aggregation and begin to be surrounded by "skirts" or "puddles" of V_2O_5; by 40 min, the particles are fully converted into V_2O_5 with a shape of low, rounded "droplets" or "puddles". At 600°C, particles are fully V_2O_5 by even 10 min.

In doped samples, on the other hand annealing at 500°C for any amount of time does not lead to dewetting, but only grain coarsening. Significant dewetting is not observed until 600°C, 20 min, conditions under which undoped films would be completely oxidized. Also, at high temperatures and long times, both doped and undoped samples develop a rough film on revealed areas of the substrate (between nanoparticles), which we attribute to oxidation of the substrate

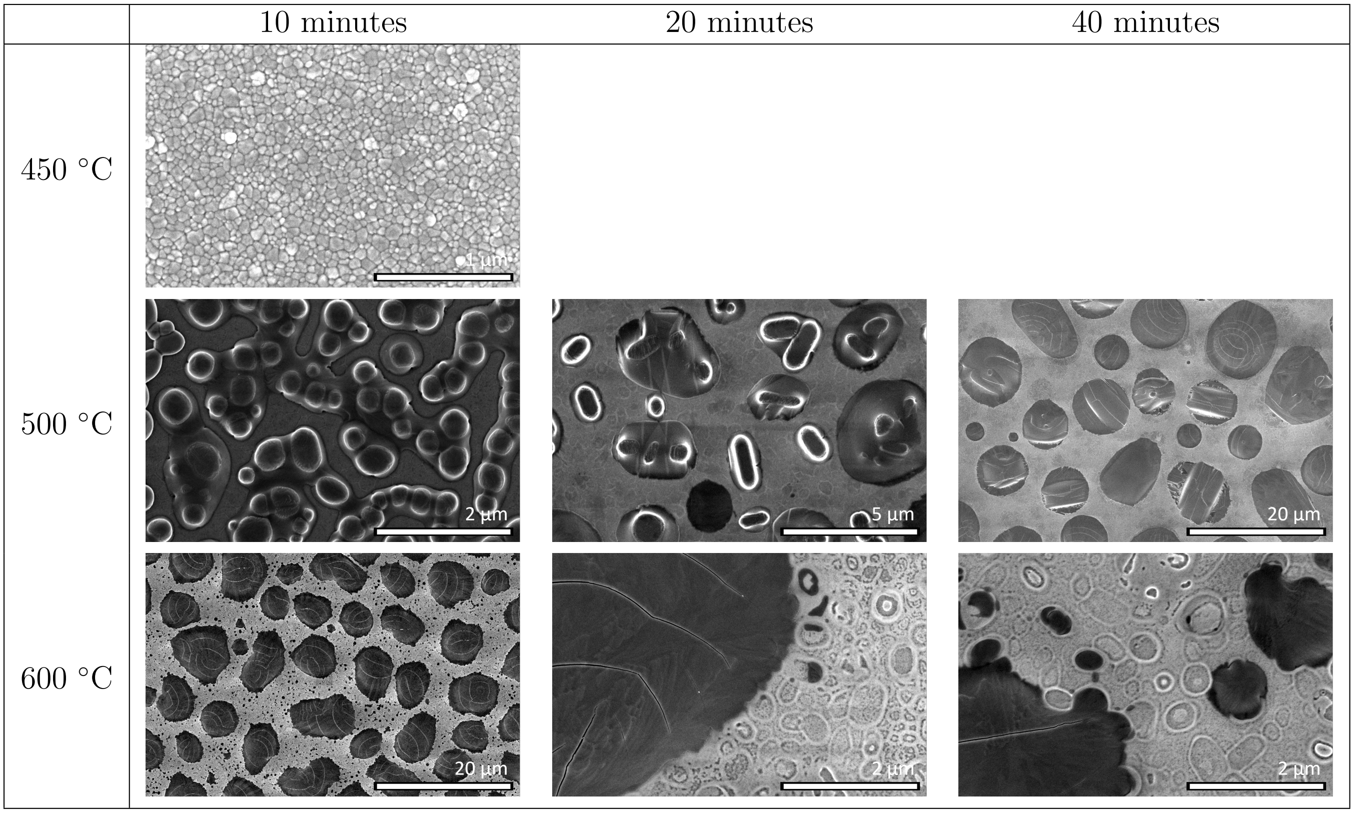

Figure S5: Undoped VO_2 annealed, from top to bottom, at 450, 500, and 600 °C, and from left to right, for 10, 20, and 40 minutes. Dewetting is first observed at 500°C, 10 min, and particles are fully oxidized by 500°C, 40 min.

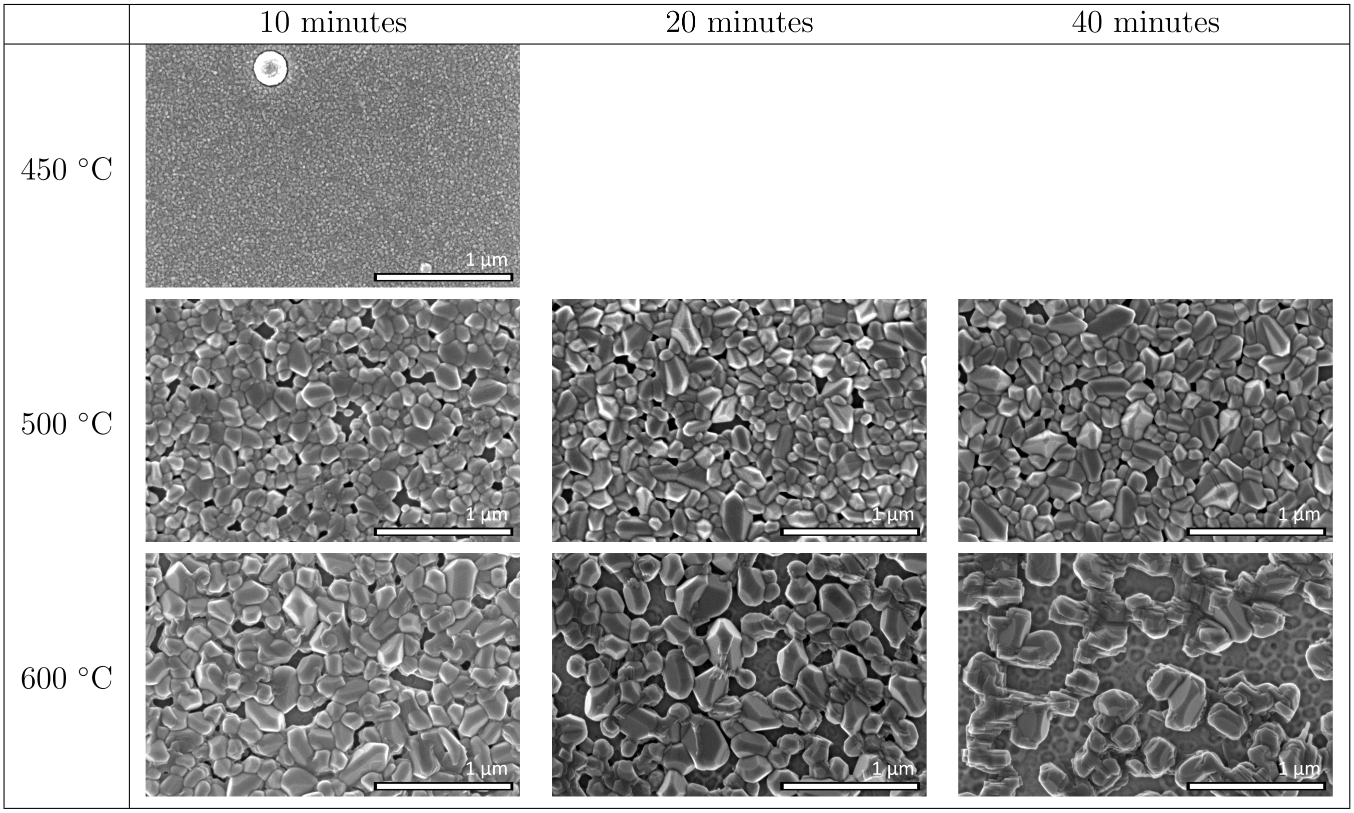

Figure S6: 5 wt.% VO_2 annealed, from top to bottom, at 450, 500, and 600 °C, and from left to right, for 10, 20, and 40 minutes. No significant dewetting is observed below 600°C, 20 min.

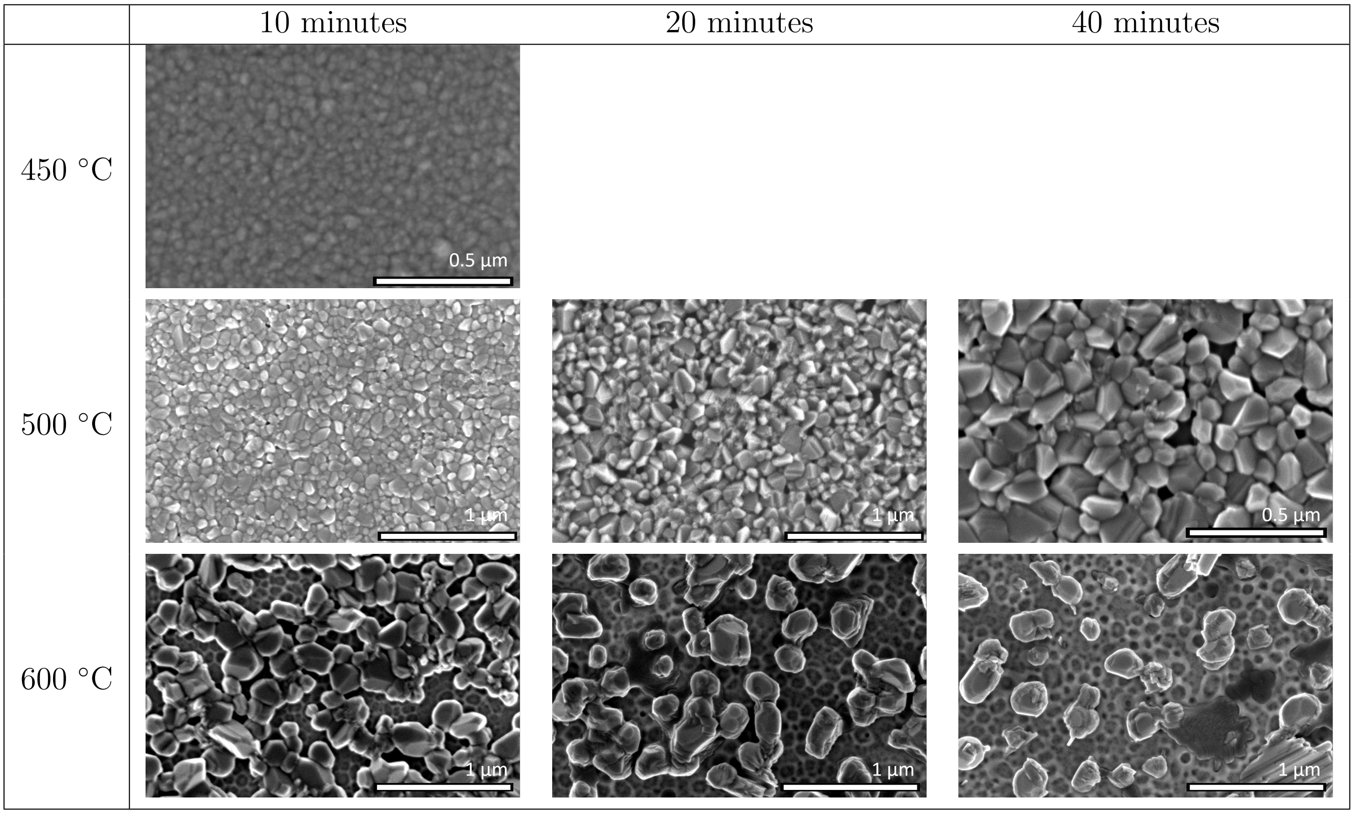

Figure S7: 8 wt.% VO_2 annealed, from top to bottom, at 450, 500, and 600 °C, and from left to right, for 10, 20, and 40 minutes. Dewetting is first obvserved at 600°C, 10 min.

S5: Another species formed at high temperatures

When annealed at high temperatures $\sim 600°C$, long, flat, crystalline sheets or platelets can be observed, in addition to the low droplets (believed to be V_2O_5) which are exemplfied in section S4. An example of such platelets are shown in Figure S8a. Similar structures can also be observed in doped films annealed at high temperatures, though here some W-VO_2 nanoparticles coexist with the platelets (Figure S8b). Like the nanoparticles, these platelets contain vanadium, oxygen, and tungsten, as shown by the EDS spectrum in Figure S8c (the occurrence of EDS peaks for K, Ca, Cu, Mg, and Al arise due to contamination on the TEM grid used for this measurement, and were observed in all measurements made using that grid). These platelets are most likely one or more of the many different vanadium oxides.

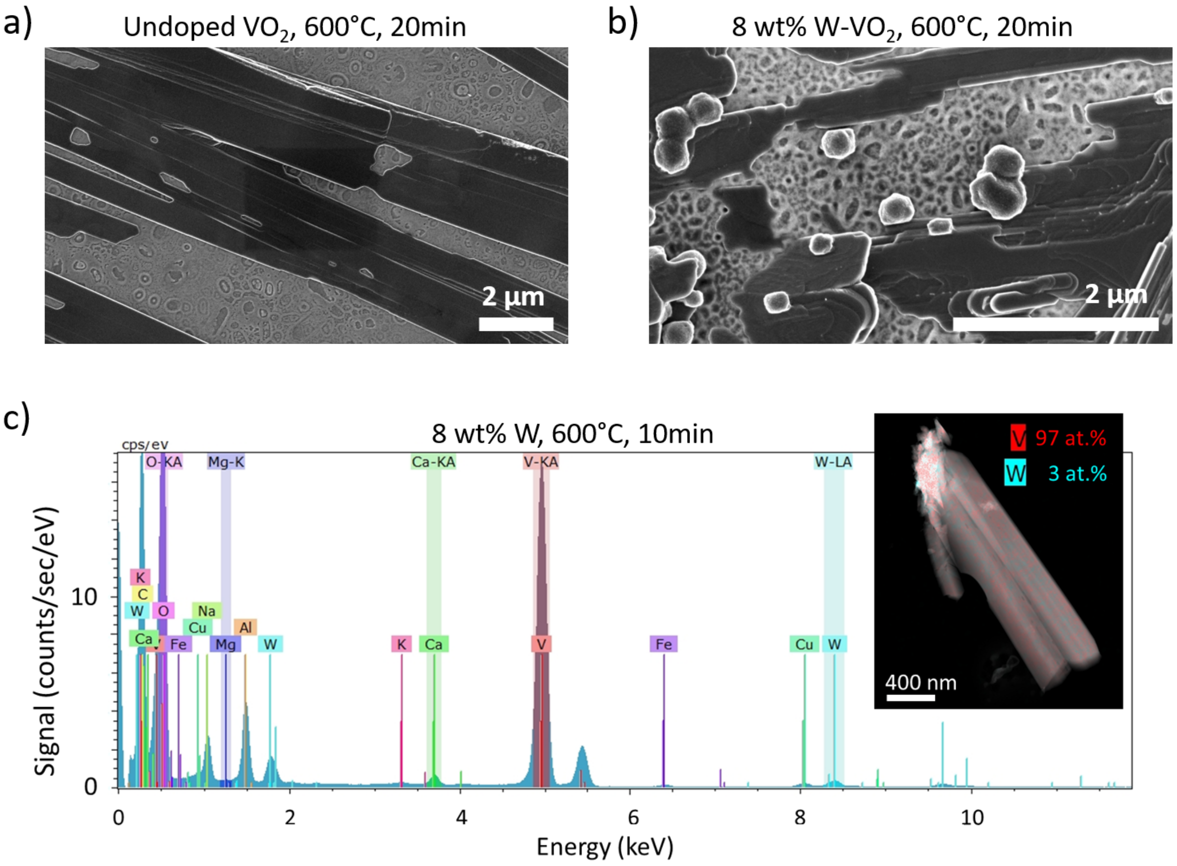

Figure S8: Both undoped films (a) and doped films (b) show the growth of platelet-like crystals in some places at high anneal temperatures. Like the nanoparticles, these are also composed of vanadium, tungsten, and oxygen.

S6: Additional TEM data

Energy dispersive spectroscopy (EDS) performed in a transmission electron microscope (TEM) allows for determination of the atomic species present in our nanoparticles. Figure S9 compares EDS spectra for an individual undoped (yellow) and W-doped (purple) nanoparticle. In both samples, clear peaks are identified corresponding to V and O (from the VO_2), and to C and Cu (from the TEM grid). In the W-doped sample, additional peaks appear corresponding to W, as expected. By fitting the EDS spectra, the relative amounts of each element can be quantitatively determined, with the exception of V and O which have significant spectral overlap making them hard to deconvolve. Thus, we can accurately measure the W:V ratio, but not the V:O ratio. Averaging across 10 separate EDS maps (each containing one or more nanoparticles), we measure the W:V ratio to be 3.3 ± 0.3 : 97 ± 3 (average$\pm 3\sigma$), which is noticeably larger than the 2.4:97.6 ratio in the original sputtering target,

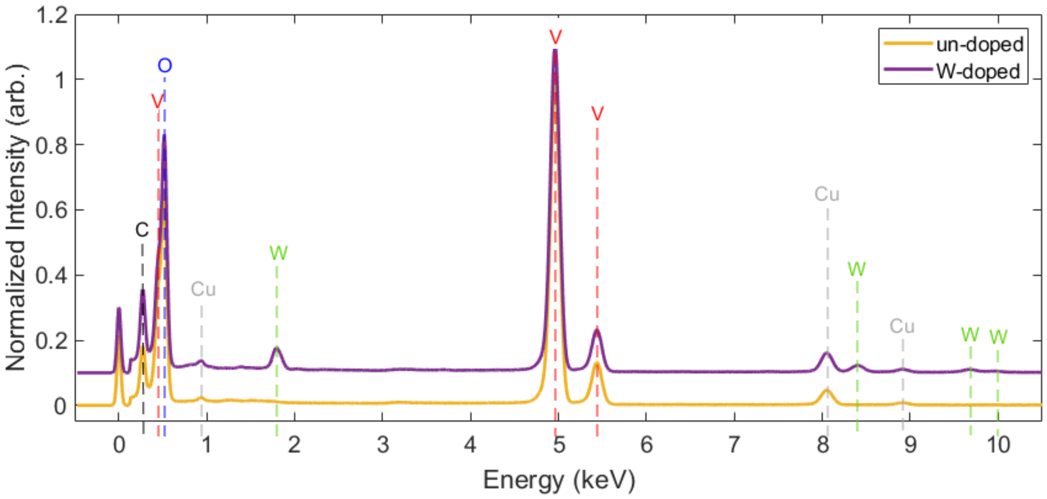

Figure S9: EDS spectra confirm the presence of W in the doped nanoparticle (purple) as opposed to the undoped (yellow).

Furthermore, EDS maps show the distribution of atoms withing the nanoparticles with sub-nm spatial resolution. Figure S10 shows four EDS maps for doped nanoparticles (a-d) and one for an undoped nanoparticle (e). As pointed out in the main text, linescans (marked

by yellow boxes along the direction of the grey arrow) show that the tungsten concentration is highest within the first few nm of the nanoparticle edges (marked by broken yellow lines), but is lower and constant throughout the bulk of the particle. On the other hand, no tungsten signal is observed within the undoped nanoparticle, as expected. Note that random noise, amplified by the calculation of atomic%, results in nonzero signal for all elements outside the particle boundaries. Although we cannot rely upon these measurements for accurate values of the O:V ratio, it is interesting to note that in each case the atomic% O is also higher at the edges and lower inside the particles. This behavior shows most strongly in the undoped nanoparticle. This is likely due to oxidization of the outside of the particle as annealing progresses.

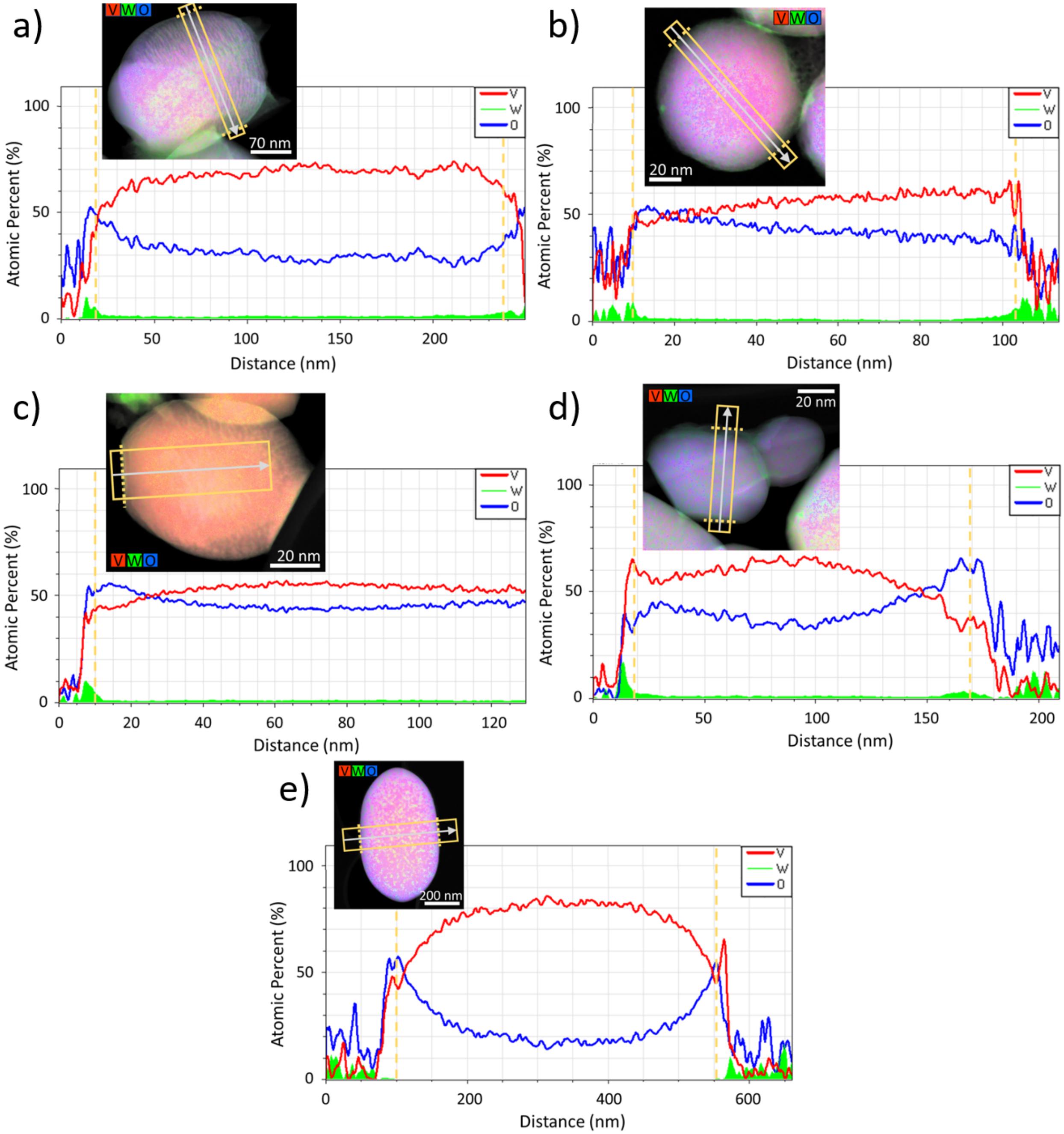

Figure S10: Linescans taken from EDS maps (insets) for doped (a-d) and undoped (e) nanoparticles show that W and O both are present in higher concentrations at the nanoparticle surface.

S7: Additional Hysteresis data

Figure S11 shows optical reflectance hysteresis measurements for undoped samples at progressively longer anneal times. At short times (when the samples are still polycrystalline films), the hysteresis loops are more narrow, with a two-step transition on the cooling curve (due to simultaneously sampling regions that have coarsened to different degrees). Once dewetting has occurred (∼10 min), the hysteresis loop becomes much broader, as discussed in the main text. As aggregation continues and the particles grow larger (approaching the size scale of visible/near-IR light), contrast decreases, due partly to increased scattering. At long times (≥20 min), contrast is further decreased by oxidization of VO_2 into V_2O_5.

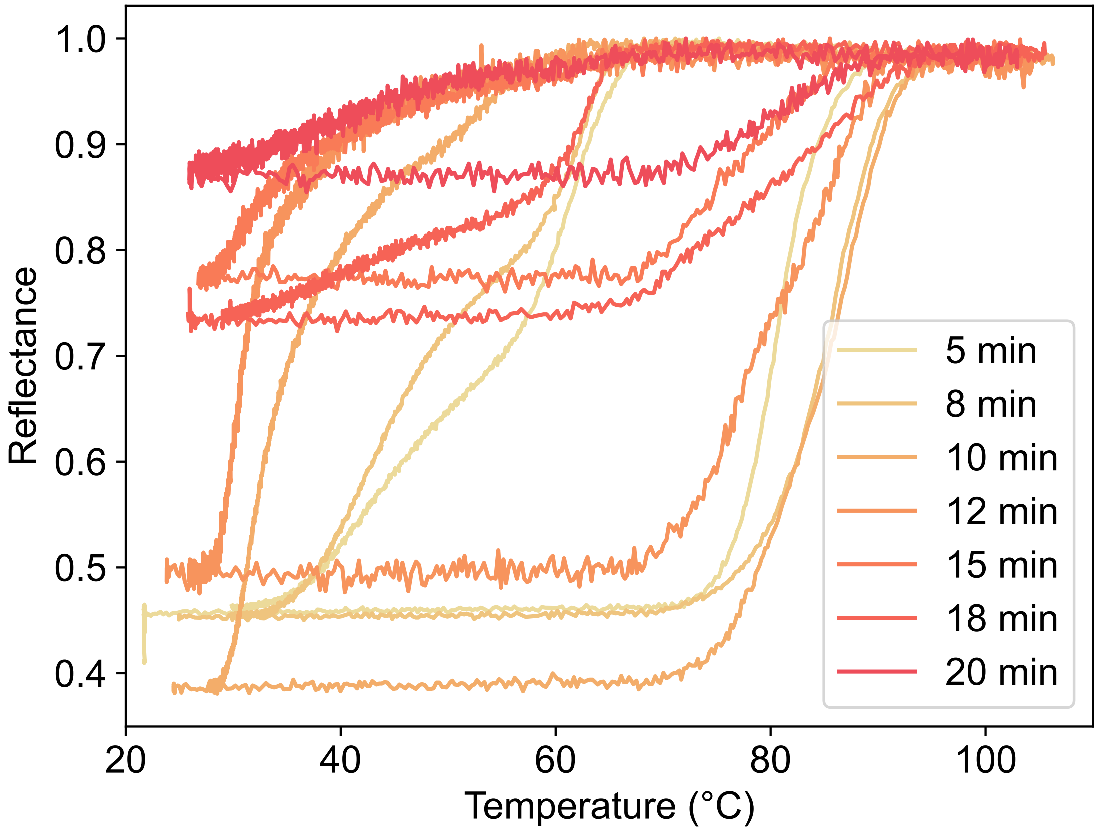

Figure S11: Hysteresis loops for undoped samples, as anneal time increases, first show an increase in hysteresis width (due to the formation of nanoparticles) and then a decreased contrast (due to scattering and formation of V_2O_5).

Figure S12 shows unnormalized versions of the hysteresis data from the main text Figure 5, representing actual percent reflection relative to a gold reference film. In addition to the features discussed in the main text, it is evident that the nanoparticulate samples (blue and orange curves) have significantly decreased reflection in both the hot and cold states. This is due to increased scattering as the particle size is on the order of the wavelength of light used for these measurements.

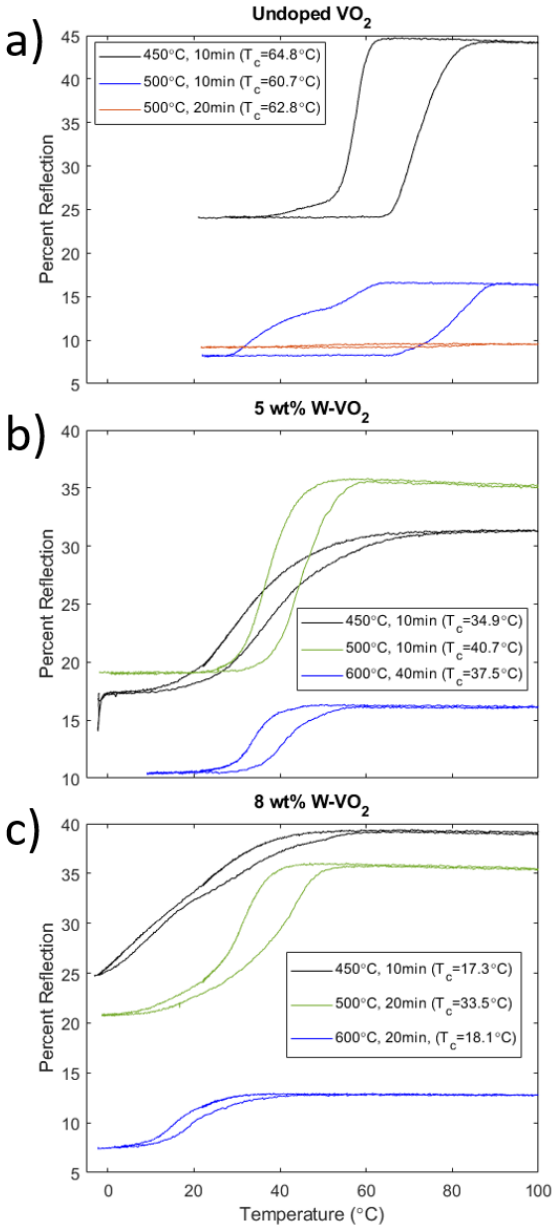

Figure S12: unnormalized reflection hysteresis loops for undoped and W-doped VO_2 films (black and green) and nanoparticles (blue and orange), showing decreased refletion from nanoparticulate samples due to scattering.

S8: Raman spectroscopy to indentify particle species

Figure S13 shows measured Raman spectra for representative samples of nanoparticles at different stages of dewetting/oxidation. The typical nanoparticles, which form at 500°C and intermediate annealing times (compare the left and central panels of the middle row of S5) show clear signatures of M1 phase VO_2 (S13, blue curves) without clear signs of other vanadium oxides present. The larger, flatter droplets that form at longer times or higher temperatures (compare the rightmost panel of the middle row and the bottom row of S5) are clearly composed of V_2O_5 (S13, orange curves). The ”skirt” that forms around the nanoparticles under intermediate conditions is likely to be amorphous V_2O_5, though other vanadium oxides may be present.

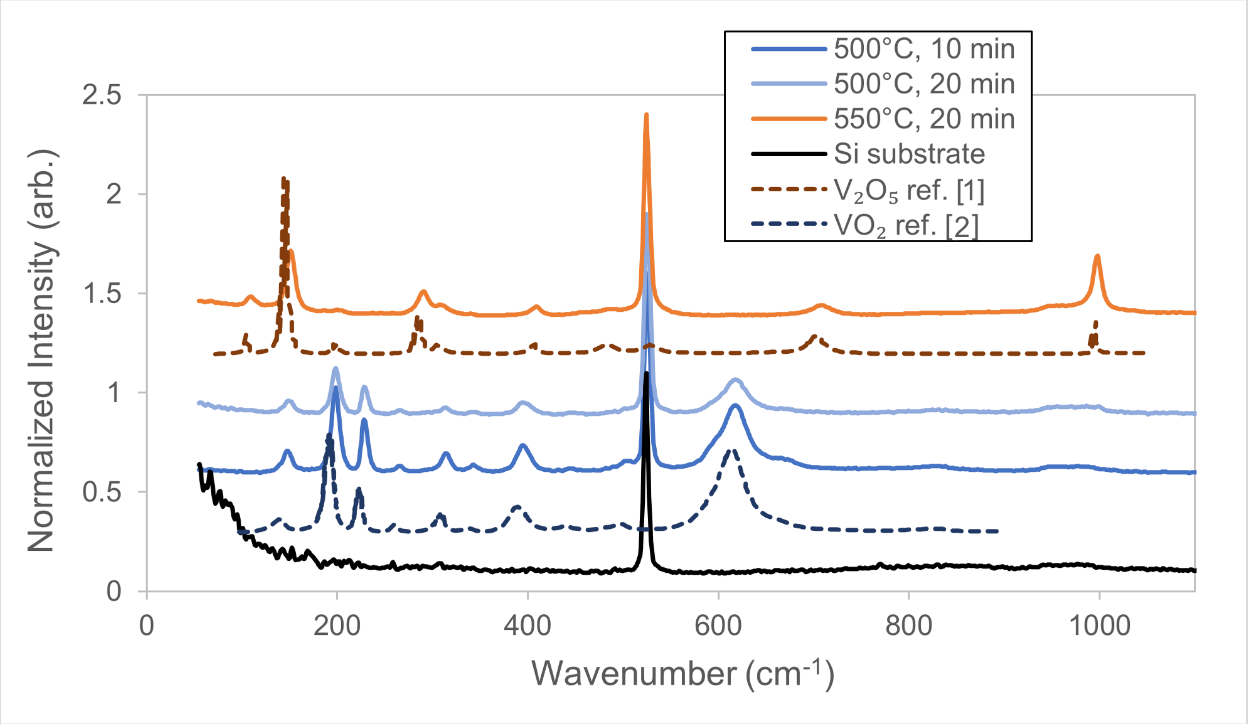

Figure S13: Raman spectroscopy confirms the presence of VO_2 in nanoparticles formed under gentler anneal conditions (blue curves) and the presence of V_2O_5 at harsher anneal conditions (orange curve). Reference spectra (broken curves) drawn from the literature[2,3] are shown for comparison.

S9: Changes in switching performance after 19 months

Figure S14 shows hysteresis spectra for a subset of the samples investigated in Figure 5 of the main text, repeated after about 19 months of storage in ambient conditions. Qualitatively, the hysteresis curves are very similar, but slight changes in contrast and T_c are reported in Table S2. For each sample, the percent contrast decreased slightly, likely due to oxidation of the VO_2 over time. In each case, the loss of contrast is worse for nanoparticles than for films, probably due to their increased surface area to volume ratio. Moreover, in each case the W-doped samples retain contrast better than the corresponding undoped samples, which could be a sign of the increased stability we hypothesized in the main text. However, the changes observed are small and a more thorough investigation over longer time scales is needed to definitively show the longevity of undoped vs doped samples. The measured transition temperatures (T_c) also differ slightly after aging, but there are no obvious trends and these discrepancies are likely within experimental uncertainty arising from changes to the experimental setup and slight spot-to-spot nonuniformities in each sample.

Table S2: Comparison of hysteretic behavior of select samples before and after 19 months of aging.

	undoped film	undoped nanoparticles	5 wt% film	5 wt% nanoparticles
Contrast (%)				
Mar 2022	45	49	45	35
Oct 2023	38	41	41	29
Difference (%)	-7	-8	-4	-6
T_c (°C)				
Mar 2022	65	63	41	38
Oct 2023	64	67	42	38
Difference (°C)	-1	4	1	0

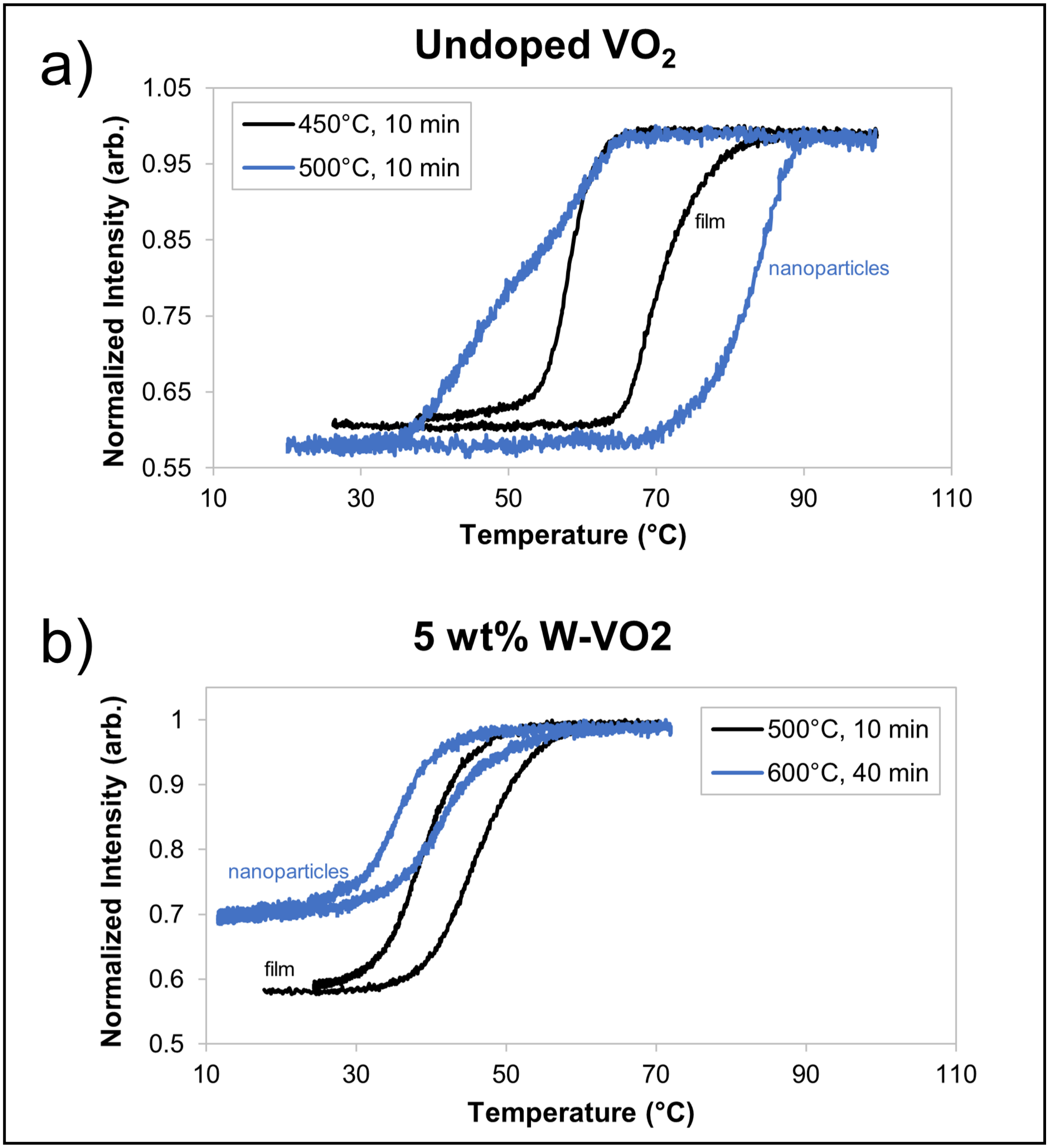

Figure S14: Hysteresis curves for undoped (a) and doped (b) VO_2 films (black) and nanoparticles (blue) after 19 months of storage show slight changes due to aging.

References

(1) Wang, L.; Ren, H.; Chen, S.; Chen, Y.; Li, B.; Zou, C.; Zhang, G.; Lu, Y. Epitaxial Growth of Well-Aligned Single-Crystalline VO_2 Micro/Nanowires Assisted by Substrate Facet Confinement. *Cryst. Growth Des.* **2018**, *18*, 3896–3901.

(2) Shvets, P.; Dikaya, O.; Maksimova, K.; Goikhman, A. A review of Raman spectroscopy of vanadium oxides. *J. Raman Spectrosc.* **2019**, *50*, 1226–1244.

(3) Baddour-Hadjean, R.; Smirnov, M. B.; Smirnov, K. S.; Kazimirov, V. Y.; Gallardo-Amores, J. M.; Amador, U.; Arroyo-de Dompablo, M. E.; Pereira-Ramos, J. P. Lattice Dynamics of β-V_2O_5: Raman Spectroscopic Insight into the Atomistic Structure of a High-Pressure Vanadium Pentoxide Polymorph. *Inorg. Chem.* **2012**, *51*, 3194–3201, PMID: 22360539.
